# Mapping and Characterization of Target-Site Resistance to Cyclic Ketoenol Insecticides in Cabbage Whiteflies, *Aleyrodes proletella* (Hemiptera: Aleyrodidae)

**DOI:** 10.3390/insects15030178

**Published:** 2024-03-06

**Authors:** Viola Müller, Frank Maiwald, Gudrun Lange, Ralf Nauen

**Affiliations:** 1 Bayer AG, Crop Science Division, R&D, Pest Control, 40789 Monheim, Germany; viola.mueller@envu.com (V.M.); frank.maiwald@bayer.com (F.M.); 2 University of Bonn, INRES, 53115 Bonn, Germany; 3 Bayer AG, Crop Science Division, R&D, 65926 Frankfurt, Germany; gudrun.lange@bayer.com

**Keywords:** insecticides, spiromesifen, spirotetramat, resistance, whiteflies, acetyl-CoA carboxylase, target-site mutation, genotyping

## Abstract

**Simple Summary:**

Cabbage whitefly (*Aleyrodes proletella*) is a destructive sucking insect pest of brassica crops, particularly white cabbage and kale. Its importance has been increased over the last decade in many geographic regions, particularly in European countries. The control of cabbage whiteflies largely relies on the application of synthetic insecticides to protect yield if populations reach economic damage thresholds. One class of insecticides to control this pest are cyclic ketoenols targeting acetyl-CoA carboxylase (ACC), an enzyme involved in fatty acid biosynthesis. In 2019, reduced efficacy of ketoenol insecticides at recommended label rates were reported. Subsequently, we collected field samples of *A. proletella* in different European countries and confirmed the presence of ketoenol resistance in laboratory bioassays. The resistance allele was shown to be an autosomal dominant trait in crossing experiments between susceptible and resistant individuals. RNA sequencing and subsequent analysis revealed a mutation, an amino acid substitution, at the ketoenol binding site in ACC. The mutation has been previously functionally validated to confer high levels of ketoenol insecticide resistance in cotton whiteflies, too. A molecular screening of 49 populations revealed the presence of the mutations in several countries. We recommend the implementation of resistance management strategies for sustainable cabbage whitefly control.

**Abstract:**

Cabbage whitefly, *Aleyrodes proletella* L., is an invasive hemipteran pest of cruciferous plants, particularly field brassica crops. Its importance has been increased over the last decade, particularly in European countries. The control of cabbage whiteflies largely relies on the application of synthetic insecticides, including tetronic and tetramic acid derivatives such as spiromesifen and spirotetramat (cyclic ketoenol insecticides), acting as insect growth regulators targeting acetyl-CoA carboxylase (ACC). In 2019, reduced efficacy against cabbage whiteflies of ketoenol insecticides at recommended label rates has been reported. Subsequently we collected field samples of *A. proletella* in different European countries and confirmed the presence of ketoenol resistance in laboratory bioassays. Reciprocal crossing experiments revealed an autosomal dominant trait, i.e., heterozygotes express a fully resistant phenotype. Transcriptome sequencing and assembly of ACC variants from resistant strains revealed the presence of an ACC target-site mutation, A2083V, as previously described and functionally validated in *Bemisia tabaci* (A2084V in *A. proletella*). Next, we employed a molecular genotyping assay to investigate the geographic spread of resistance and analyzed 49 populations collected in eight European countries. Resistance allele frequency was highest in the Netherlands, followed by Germany. Finally, we provide a proposal for the implementation of appropriate resistance management strategies.

## 1. Introduction

Cabbage whitefly, *Aleyrodes proletella* L. (Hemiptera: Aleyrodidae), is an invasive and emerging sucking pest species originating from Europe [1,2]. It has been spread globally and is found in different geographies including for example China [3,4], India [5], Africa, North America, and Australia [6]. It is primarily a pest on cruciferous crops, with a strong preference for cabbage, cauliflower, kale, Brussels sprouts, and broccoli [1,7]. Originally it was a minor pest, but outbreaks on Brassica crops especially in Europe has rendered it a major problem in horticultural production systems in recent years [7,8,9,10]. Cabbage whitefly is not known as a virus vector [11], but causes damage to Brassica crops by direct feeding and the excretion of honeydew serving as a substrate for sooty mold affecting plant quality [8]. After overwintering, adult females lay eggs on suitable host plants and depending on temperature a typical lifecycle from egg hatch to imago is completed within 3–6 weeks [8,12]. Three to five generations per season were reported in the UK [13], but up to 10 generations seem possible under ideal conditions in southern Europe [14].

Cabbage whitefly control largely relies on the application of insecticides to keep infestation levels under economic damage thresholds [15], but integrated approaches including biological control measures [16,17], resistant varieties and agricultural practices have been promoted recently [18,19,20]. Different chemical classes of insecticides have been shown to provide efficacy against different life stages of *A. proletella*, including neonicotinoids, pyrethroids, and cyclic ketoenols such as spiromesifen and spirotetramat [15,21]. Neonicotinoids such as imidacloprid and thiamethoxam performed best by drench application treatments [21], whereas spirotetramat has been reported to be among those insecticides most effective against cabbage whiteflies upon foliar application [15]. Cyclic ketoenols, also known as tetramic and tetronic acid derivatives [22,23], are acaricidal and insecticidal lipid biosynthesis inhibitors, particularly active against juvenile stages of various sucking pest species [23,24]. This class of chemistry currently comprises four commercial compounds: spirodiclofen, spiromesifen, spirotetramat, and spiropidion. All of them except spirodiclofen are active against major whitefly pests such as *Bemisia tabaci*, *Trialeurodes vaporariorum* und *A. proletella* [25,26,27]. Ketoenols act as insect growth regulators and target acetyl-CoA carboxylase (ACC) by binding to the carboxyltransferase (CT) domain of this multi-domain enzyme which is encoded by a single gene in insects [28], and known to catalyze the first committed step in fatty acid biosynthesis, i.e., the carboxylation of acetyl-CoA to malonyl-CoA [29].

Spiromesifen and spirotetramat were introduced to the market in 2004 and 2008, respectively. Since then, they have been globally used for whitefly control, and in some regions, continuous selection led to the evolution of moderate to high resistance levels in cotton and greenhouse whiteflies, *B. tabaci* and *T. vaporariorum*, respectively [30,31]. Ketoenol resistance in *B. tabaci* collected in Spain and Australia has been reported to be conferred by an ACC target-site mutation, A2083V, in the CT domain of the enzyme. The strong impact of the A2083V mutation on ketoenol efficacy has been demonstrated and functionally validated in bioassays with transgenic *Drosophila* lines [32], and transgenic *Caenorhabditis elegans* [33]. Recent resistance monitoring campaigns utilizing molecular diagnostics revealed the presence of this mutation in Australian and Greek *B. tabaci* greenhouse and field populations, respectively [34,35]. Another target-site mutation, originally described in spiromesifen resistant *T. vaporariorum*, E645K [30], is located outside the CT domain, and its association with ketoenol resistance phenotypes was not confirmed [36]. Indeed, attempts to introduce the mutation in *Drosophila* using CRISPR/Cas9 genome editing failed as it turned out to be homozygous lethal [32]. Metabolic resistance mechanisms driven by detoxification enzymes such as cytochrome-P450 monooxygenases (P450s), glutathione S-transferases, carboxylesterases, and UDP-glycosyltransferases have not yet been described in whiteflies [37], but these have been shown to play a major role in spider mites, e.g., *Tetranychus urticae*, targeted by ketoenol insecticides [38,39]. Despite increasing selection pressure no cases of insecticide resistance have yet been described in *A. proletella*, except for pyrethroids in the UK [40].

In 2019 and 2020, we collected cabbage whitefly field populations from white cabbage that survived ketoenol insecticide treatments at recommended label rates in Belgium and Germany, respectively. As it was uncertain if the lack of field efficacy was due to application issues or resistance, we started to investigate the ketoenol susceptibility in field collected cabbage whitefly strains from various locations in Europe. As a second step, when ketoenol resistance was confirmed, we aimed to characterize the genetics and molecular mechanisms of resistance with special reference to an ACC target-site mutation previously reported in *B. tabaci*. Finally, we developed a genotyping diagnostic for resistance monitoring purposes to support the implementation of resistance management strategies for sustainable *A. proletella* control.

## 2. Materials and Methods

### 2.1. Insects

Ten populations of *A. proletella* were collected in different European countries in 2019 and 2020 and reared under greenhouse conditions on untreated savoy cabbage plants *Brassica oleracea* L. var. capitata at 23 ± 1 °C, 50% relative humidity, and a photoperiod of L16:D8 (Table 1). Three field strains, SPI-5/19, SPI-2/20, and SPI-6/20 were kept under constant selection pressure on cabbage plants treated with 200 mg L^−1^ (a.i.) spiromesifen by spray application. After two generations, the selected strains were stable and no longer affected by concentrations of 200 mg L^−1^ of both spiromesifen and spirotetramat. Strain 1/19 was discontinued after we conducted spiromesifen and spirotetramat bioassays (incl. genotyping by pyrosequencing). The most susceptible strain of each insecticide bioassay was chosen as a reference to calculate resistance ratios (RR).

In addition, we collected 39 *A. proletella* populations from various countries between 2019 and 2021 which were preserved in 70% (*v*/*v*) ethanol and stored at 4 °C until further analysis by pyrosequencing (Table S1).

### 2.2. Chemicals

Proprietary commercial formulations of spiromesifen, Oberon^®^ SC 240, and spirotetramat, Movento^®^ OD 150 (Bayer CropScience, Monheim, Germany), were used and diluted with tap water as needed. Acetamiprid and *λ*-cyhalothrin were of analytical grade and purchased from Sigma Aldrich (St. Louis, MO, USA). Trizol Reagent was provided by Invitrogen, ThermoFisher Scientific (Waltham, MA, USA). HPLC gradient grade solvents were purchased from Merck (Darmstadt, Germany). Unless otherwise stated, all other chemicals were of analytical grade and obtained from Sigma-Aldrich (St. Louis, MO, USA).

### 2.3. Nymph Bioassays

Whitefly nymph bioassays were conducted as previously described by Lueke et al. [32] with minor modifications. Briefly: Two-week-old cabbage plants (*B. oleracea* L. var. capitata) were trimmed to two true leaves per plant, each leaf representing one replicate. In total, two cabbage plants (4 replicates) were used per insecticide concentration tested. Two cabbage plants were infested with approx. 100 adult whiteflies for 24 h. Afterwards, the adults were removed, and the plants kept under greenhouse conditions at 24 ± 1 °C, 50% relative humidity and a photoperiod of L16:D8 for 10–13 days to allow for 2nd-instar development. The infested plants were treated with serial dilutions of aqueous insecticide solutions using a purpose-built in-house spraying device. Spiromesifen and spirotetramat formulations were applied at concentrations of 0.32 to 200 mg L^−1^ (a.i.) in aqueous 0.02% (*w*/*v*) Triton X-100. Control plants were treated with aqueous 0.02% (*w*/*v*) Triton X-100 only. Ten days after insecticide application, all leaves were evaluated for dead and affected whitefly nymphs, i.e., those not developed to 4th instar/puparia. All bioassays were replicated twice.

### 2.4. Adult Bioassays

Acetamiprid and λ-cyhalothrin are fast-acting neurotoxic insecticides and were tested against *A. proletella* adults using a leaf-disc dip assay. Leaf discs (30 mm in diameter) of two-week-old *B. oleracea* L. var. capitata plants were dipped for 3 s in aqueous insecticide solutions (concentration range 0.128 to 2000 mg L^−1^; in triplicate) diluted with 0.02% (*w*/*v*) Triton X-100. The leaf discs were air-dried on a filter paper for approximately 20 min and then placed with their adaxial surface downwards onto a bed of agar (15 g L^−1^) in 6-well tissue culture plates (Greiner Bio-One GmbH, Frickenhausen, Germany). Leaf discs treated with 0.02% (*w*/*v*) aqueous Triton X-100 served as control. *A. proletella* adults were collected from rearing cages by means of a vacuum-pump powered aspirator and after brief CO_2_ anesthesia, 20 adults were placed onto the leaf-discs. Afterwards, each 6-well plate was sealed with a ventilated porous foil and stored upside down. Whiteflies were scored for mortality after 72 h. Each bioassay including three replicates per concentration was replicated twice.

### 2.5. Reciprocal Crossing Experiments

Reciprocal crossing experiments with two *A. proletella* strains were conducted as previously described for *B. tabaci* with minor modifications [32]. Briefly: Leaf discs with pupae (4th instar) close to adult emergence were placed on non-infested cabbage leaf discs in Petri dishes incl. a wetted filter paper disc (90 mm in diameter). Two strains were selected for reciprocal crossing experiments, i.e., strains 6/19 (susceptible) and SPI-5/19 (resistant and kept under selection pressure with 200 mg L^−1^ spiromesifen). To obtain unmated individuals for the reciprocal crosses, we separated pupae prior to adult emergence. Sex of pupae was determined by size [41], with male pupa (haploid) smaller than female (diploid). Female and male pupae of both strains were segregated as described previously [32]. Individual males and females were paired on cabbage leaf discs in Petri dishes to obtain the following F1 progeny: SPI-5/19 ♀ × ♂ 6/19 and 6/19 ♀ × ♂ SPI-5/19. Seven days after crossing the leaves were transferred from the Petri dishes to three-week-old cabbage plants. After 6 days, the leaf disc was removed and cabbage plants infested with F1 progeny of reciprocal crosses were treated with a discriminating rate of 200 mg L^−1^ (a.i.) spiromesifen (Oberon 240SC) by spray application as described above. Ten days later, nymphs were scored for mortality. The experiment was replicated four times and mean mortality ± SD at the applied discrimination rate was calculated for the reciprocal crosses along with the parental strains, SPI-5/19 and 6/19 tested in parallel. The level of dominance was calculated according to Liu and Tabashnik [42].

### 2.6. RNA Sequencing and Acetyl-CoA Carboxylase Assembly

The susceptible *A. proletella* strains 3/19, 4/19, 6/19, and 5/20 as well as the ketoenol resistant populations 2/20 and SPI-2/20 were selected for an RNAseq approach. RNA of ten *A. proletella* adults per replicate was extracted with Trizol reagent (ThermoFisher Scientific, Waltham, MA, USA). Insects were flash frozen in liquid nitrogen and homogenized with two 3 mm steel beads at 20 Hz for 2 × 10 s with a MM300 laboratory bead mill (Retsch GmbH, Haan, Germany). After Trizol was added to the crushed insects, the samples were incubated for 5 min at room temperature. Then, 100 µL chloroform was added before samples were inverted for 15 s and incubated for three additional minutes. After a centrifugation step of 15 min at 10,000× *g* and 4 °C, the aqueous phase was used for RNA purification with RNAdvance kit (Beckman Coulter, München, Germany) according to the manufacturer’s instructions. The quality and concentration of RNA was determined using the Infinity M200Pro plate reader (Tecan Trading AG, Männedorf, Switzerland). After preparation of the strand-specific cDNA library, samples were subjected to Illumina sequencing (5 Mio reads, 2 × 150 bp). Afterwards, all sequencing data reads of the four replicates per strain were assembled using Trinity 2.8.5 [43] and further consolidated with TransDecoder 5.3.0 (https://github.com/TransDecoder, accessed on 16 February 2024) using blast results versus SwissProt 2021_4. *A. proletella* ACC sequences were identified by selecting full length blast hits using *B. tabaci* QJQ31013.1 as the query. The *T. vaporariorum* ACC sequence was constructed using an alignment of *B. tabaci* coding sequence (MN567040.1) vs. contig VMOF01000024.1 of the genomic assembly ASM1176424v1 by GMAP as a guideline [44]. Multiple alignment was performed using Clustal omega v.1.2.3 [45]. *A. proletella* RNAseq data and assembled ACC genes have been submitted to GenBank and are accessible under BioProject PRJNA832135. The ACC genes can be accessed via GenBank under GJYF01020973.1 (strain SPI-2/20), GJYF01046828.1 (3/19), GJYF01092380.1 (4/19), GJYF01105771.1 (5/20), GJYF01137039.1 (6/19), GJYF01139669.1 (*T. vaporariorum*), XP_022181497.1 (*Myzus persicae*) and QJQ31013.1 (*B. tabaci*).

### 2.7. RT-qPCR

RNA of ten *A. proletella* adults per replicate and strain was extracted as described above. High-quality RNA (OD 260/280 1.8–2.0 and OD 260/230 2.0–2.2) as measured by the Infinity M200Pro plate reader (Tecan Trading AG, Männedorf, Switzerland) was subjected to cDNA library preparation using the iScript synthesis kit (ThermoFisher AG, Waltham, MA, USA) according to the manufacturer’s instructions. In RT-qPCR reactions, 10 µL SsoAdvanced Universal SYBR Green Supermix (Bio-Rad, Hercules, CA, USA) was mixed with 5 µM primer dilutions (fc 0.3 µM) and 5 µL cDNA (Table S2). For *ACC* expression analysis, 8 ng/µL cDNA was used. The reference genes *actin*, *HSP-90*, and *ATPase* were selected from the RNAseq analysis (BioProject PRJNA832135). The RT-qPCR reaction was started at 95 °C for 3 min, following 40 rounds at 95 °C for 15 s, and 60 °C for 30 s. A melting curve at 65–95 °C with a 0.5 °C increment for 5 s per step finalized the reaction. The whole reaction was performed in a C1000 Touch Thermal Cycler (Bio-Rad, Hercules, CA, USA). Each RT-qPCR experiment comprised four biological and three technical replicates per strain. Wells with nuclease-free water instead of cDNA served as control. Evaluation of the data was performed using CFX Maestro Software, v2.3 (Bio-Rad, Hercules, CA, USA) as well as qbase+ v2.0 (Biogazelle, Zwijnaarde, Belgium).

### 2.8. Genotyping by Pyrosequencing

The genomic DNA of individual *A. proletella* (*n* = 10) whiteflies was isolated using the DNAdvance kit (Beckman Coulter, München, Germany) according to manufacturer instructions. After quality confirmation as described above, DNA was subjected to a PCR reaction containing 25 µL in total. As polymerase served 2x JumpStart Taq Ready Mix (Sigma-Aldrich, St. Louis, MO, USA) and 10 µM primer (Table S3; Figure S1) dilutions were added to the reaction mix before the PCR was started for 3 min at 95 °C. For ACC A2083V (*B. tabaci* numbering) genotyping, 45 rounds at 95 °C for 30 s, 58 °C at 30 s, and 72 °C at 3 s followed. After the final step for 5 min at 72 °C, PCR was completed. Pyrosequencing was conducted using the PyroMark Q96 ID and PyroMark Gold Q96 Reagent Kit (Qiagen, Hilden, Germany) according to the manufacturer instructions. The resulting pyrograms were analyzed with the PyroMark Q96 ID Software 2.5 (Qiagen, Hilden, Germany).

### 2.9. Computational Modeling and Docking

Structure-based design and analysis was caried out using the X-ray co-crystal structure of the CT domain of *Saccharomyces cerevisiae* acetyl-CoA carboxylase in complex with pinoxaden (GenBank: 3PGQ) [46]. Homology modeling and the visualization of the results was performed using Maestro Schrödinger release 2018-1 (Schrödinger LLC, New York, NY, USA). Ketoenol insecticide docking and interaction analysis was performed using SeeSAR from BioSolveIT GmbH (St. Augustin, Germany) and the scoring function HYDE [47].

### 2.10. Statistical Analysis

Bioassay data were corrected for control mortality according to Abbott [48], and LD_50_-values and 95% confidence intervals (95% CI) calculated by probit analysis using PoloPlus 2.0 (LeOra Software, v2.0, Petaluma, CA, USA). Mean percentage mortality values of discriminating dose bioassays ± SD (*n* = 4) was analyzed by Graph Pad Prism v8 (GraphPad Software, Boston, MA, USA). The level of dominance (*h*) of ketoenol resistance was calculated from mean mortality figures of reciprocal crossing experiments by the single concentration method [42]. Values of *h* range from 0 (completely recessive resistance) to 1 (completely dominant resistance). Further information on statistical data analysis is given in respective figure legends where appropriate. All other experimental data were analyzed and visualized using GraphPad Prism v8 (GraphPad Software, Boston, MA, USA) unless otherwise stated.

## 3. Results

### 3.1. Bioassays

Nymph bioassays conducted with ten *A. proletella* field strains collected in 2019 and 2020 revealed ketoenol susceptibility for most strains, except 5/19 (Belgium), 2/20, and 6/20 (Germany) which exhibited resistance ratios (RR) against spiromesifen and spirotetramat between 56- and >72-fold and between 16- and >56-fold, respectively, when compared to the most susceptible field strain against both compounds (Table 2). Strains 5/19, 2/20 and 6/20 were subsequently kept under selection pressure with spiromesifen (200 mg L^−1^) resulting in strains SPI-5/19, SPI-2/20, and SPI-6/20 being completely resistant to the highest concentration tested of both spiromesifen and spirotetramat (Table 2). In summary, bioassays confirmed the presence of ketoenol cross-resistance in cabbage whitefly strains collected in Belgium and Germany in 2019 and 2020, respectively, whereas *A. proletella* strains collected in France (1/19, 4/19) and Croatia (3/19) in 2019 were susceptible to ketoenols.

Next, we tested all field-collected *A. proletella* strains (except 1/19, which was discontinued during the study) including two of the ketoenol-selected strains for cross-resistance against two insecticides with a neuronal mode of action, acetamiprid and λ-cyhalothrin, which are registered for whitefly control in many countries. Expectedly, we did not find cross-resistance between ketoenols and these neuronal insecticides representing different chemical classes and modes of action. The variation in acetamiprid and λ-cyhalothrin susceptibility between strains was rather low, i.e., 1–3-fold and 1–8-fold, respectively. In summary, acetamiprid showed a more consistent level of efficacy against cabbage whitefly adults when compared to λ-cyhalothrin (Table 3).

### 3.2. Reciprocal Crossing Experiments

**Figure 1:**
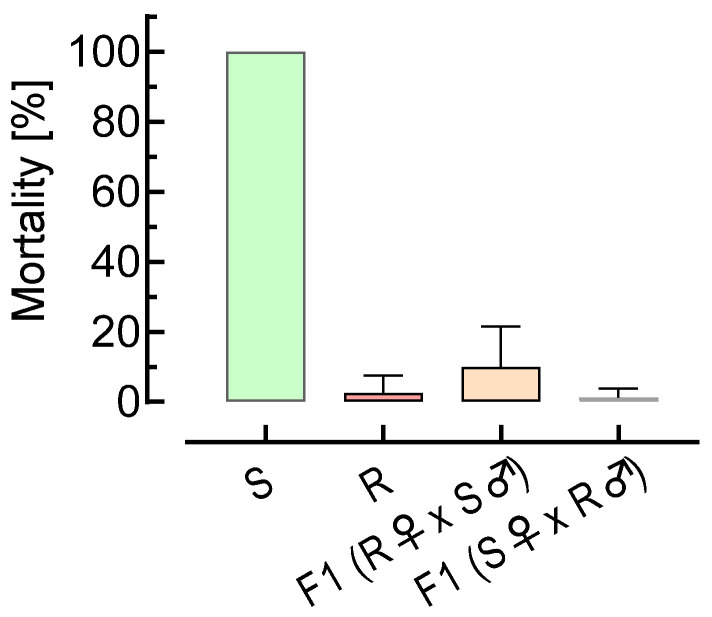
Genetics of ketoenol resistance in *Aleyrodes proletella*. Efficacy of a discriminating dose of 200 mg L^−1^ spiromesifen against 2nd-instar nymphs (F1) resulting from reciprocal crosses of *A. proletella* adults of strains 6/19 (S, susceptible) and SPI-5/19 (R, selected resistant). Data are mean values ± SD (*n* = 4).

Reciprocal crosses of Belgium strains 6/19 (susceptible) and SPI-5/19 (selected, highly resistant) and a subsequent discriminating dose bioassay with 200 mg L^−1^ spiromesifen revealed autosomal inheritance with minor differences observed in mortality between F1 progeny and SPI-5/19 (Figure 1). The estimated degree of dominance (*h*) based on combined survival (94.4%) of F1 6/19 ♀ × ♂ SPI-5/19 and F1 SPI-5/19 ♀ × ♂ 6/19 at 200 mg L^−1^ spiromesifen was 0.97, suggesting dominant resistance; i.e., heterozygotes express a ketoenol resistance phenotype.

### 3.3. RNAseq and acetyl-CoA Carboxylase (ACC) Assembly

**Figure 2:**
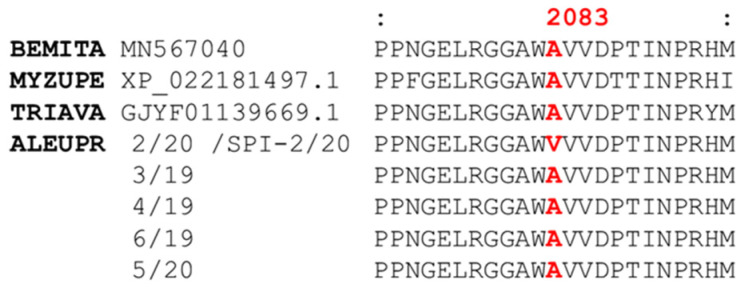
Alignment of partial acetyl-CoA carboxylase (ACC) sequences. The amino acid alignment shows the conserved region of the carboxyltransferase (CT) domain harboring the A2083V (*Bemisia tabaci* (BEMITA) ACC numbering) target-site mutation detected in field populations of *Aleyrodes proletella* such as strain 2/20 highly resistant to ketoenol insecticides. Strain SPI-2/20 originates from strain 2/20, but continuously maintained under selection pressure with 200 mg L^−1^ spiromesifen. Abbreviations: MYZUPE, *Myzus persicae*; TRIAVA, *Trialeurodes vaporariorum*; ALEUPR, *Aleyrodes proletella*. GenBank accession numbers: 2/20 (GJYF01020973.1), 3/19 (GJYF01046828.1), 4/19 (GJYF01092380.1), 6/19 (GJYF01137039.1) and 5/20 (GJYF01105771.1). A full-length ACC alignment is given in Figure S3.

Illumina RNA sequencing and subsequent analysis was conducted with six strains, including the susceptible strains 3/19, 4/19, 6/19, and 5/20 as well as the resistant strains 2/20 and SPI-2/20 (selected). All data were submitted to GenBank under BioProject No. PRJNA832135. Based on a previous study with *B. tabaci* where ketoenol resistance was shown to be conferred by an ACC A2083V target-site mutation [32], we assembled and analyzed *A. proletella* ACC sequences based on *B. tabaci* ACC (GenBank: QJQ31013.1) as the query and obtained full-length *A. proletella* ACC sequences containing 2343 amino acids for all strains analyzed (Figure S2). Indeed, we only identified a single non-synonymous mutation, resulting in A2084V, in the ACC assembly of the ketoenol resistant *A. proletella* strains 2/20 and SPI-2/20. The position is equal to A2083V in *B. tabaci* ACC, recently shown to confer ketoenol resistance by functional validation in transgenic Drosophila expressing mutant ACC [32]. For convenience we continue to refer to A2083V for the remainder of the article. The A2083V mutation is found in a highly conserved region of the carboxyltransferase (CT) domain of ACC (Figure 2).

The full-length sequences of the assembled ACCs from all *A. proletella* strains are shown in Figure S3 and were submitted to GenBank (accession numbers are given in Figure S3).

Next, we genotyped by pyrosequencing 10 adult female whiteflies per strain for the presence of the A2083V mutation (Table 4). Strains 3/19, 4/19, 6/19, 1/20, 4/20, and 5/20 were homozygous for A2083, whereas the selected strains SPI-5/19, SPI-2/20, and SPI-6/20 were mostly homozygous for V2083, with a few heterozygotes present (10–20%) and no homozygotes for A2083, thus matching the bioassay data shown in Table 2. Continuous selection pressure of strains 5/19, 2/20, and 6/20 with 200 mg L^−1^ spiromesifen resulted in the cumulation of A2083V heterozygotes and homozygotes (Table 4).

### 3.4. Acetyl-CoA Carboxylase (ACC) Expression Level

**Figure 3:**
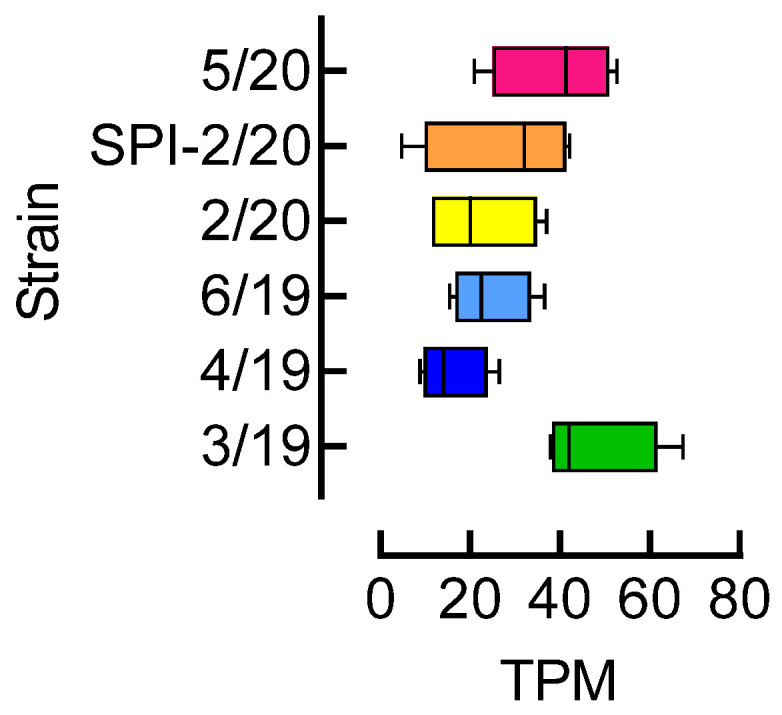
Abundance of acetyl-CoA carboxylase (ACC) transcripts in adults of different strains of *Aleyrodes proletella*. Box and whiskers plot (min to max) of ACC transcripts per million (TPM) expression values scaled to a common median of 10. Data are based on RNAseq analysis of individual adults (*n* = 4) from different strains.

ACC expression level in *A. proletella* adults of the ketoenol susceptible strains 3/19, 4/19, 6/19, and 5/20, and the resistant strains 2/20 and SPI-2/20 was analyzed based on abundance of ACC transcripts, and revealed no obvious trends linked to the observed ketoenol resistance ratios (Figure 3).

### 3.5. Mapping of Ketoenol Resistance by Analyzing Alcohol Persevered A. proletella Field Samples

**Figure 4:**
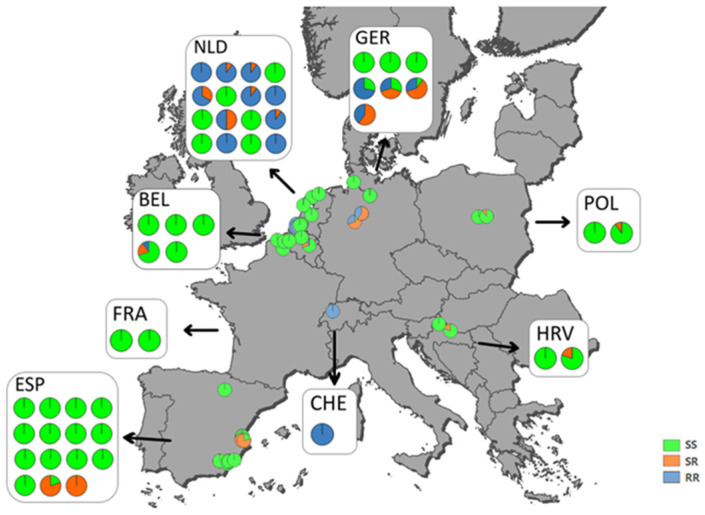
Mapping of ketoenol resistance in different European countries. Genotyping of ACC A2083V ketoenol target-site resistance alleles in adults of *Aleyrodes proletella* populations collected from eight European countries between 2019 and 2021. Pie charts display the proportion of RR resistant homozygote (blue), SR heterozygote (orange), and SS susceptible homozygote (green) genotypes. Country codes: BEL, Belgium; CHE, Switzerland; ESP, Spain; FRA, France; GER, Germany; HRV, Croatia; NLD, Netherlands; POL, Poland.

Between 2019 and 2021, we collected 49 *A. proletella* field samples in eight European countries and subjected genomic DNA of ten individuals per strain to pyrosequencing analysis for ACC resistance allele frequency. We detected the A2083V allele in 19 field samples, whereas no target-site mutation was detected in 30 field samples; i.e., all individuals in these samples were A2083 (Figure 4). Based on the genetics of ketoenol resistance (autosomal, dominant) and bioassay results obtained for the selected strains, we could suggest that at least two, five, and eleven strains collected in 2019, 2020, and 2021, respectively, would resist recommended label rates of ketoenol insecticides. The highest frequency of ketoenol resistance alleles has been detected in cabbage whitefly samples collected in the Netherlands and Germany, whereas most of the Spanish samples were homozygous susceptible A2083. Details and breakdown of the genotyping results displayed in Figure 4 are provided in Table 4 and the Supplementary Materials (Table S4).

### 3.6. Computational Modelling and Ketoenol Docking Analysis

**Figure 5:**
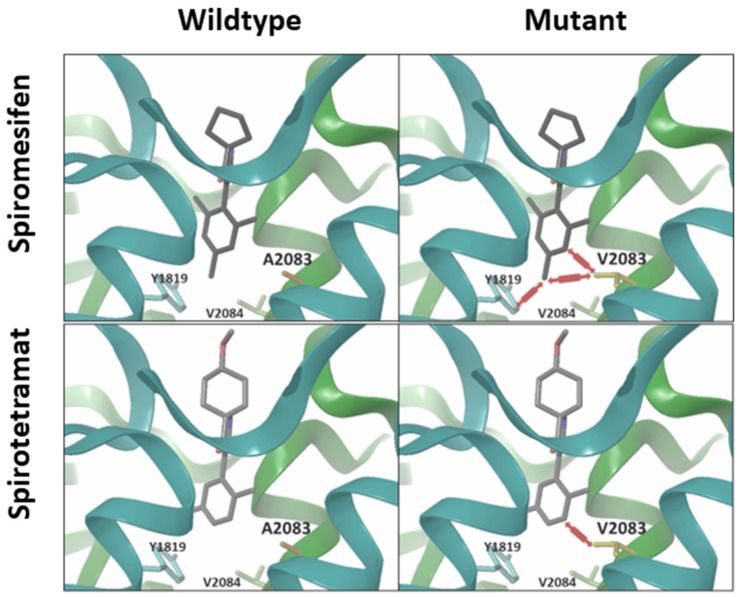
Computational modelling. Molecular docking of spiromesifen and spirotetramat into the catalytic pocket of the acetyl-CoA carboxylase (ACC) carboxyltransferase (CT) domain suggests implications for binding in the mutant form (V2083) when compared to the wild-type form (A2083). Potential clashes between ketoenols and amino acid residues interfering with high-affinity binding in the mutant variant are depicted by red arrows. The homology models are based GenBank entry 3PGQ.

Previous work has functionally validated the importance of the ACC A2083V target-site mutation for ketoenol resistance in vivo, but in silico ketoenol docking studies in wildtype and mutant ACC have not been conducted yet. Molecular fitting of spiromesifen and spirotetramat within the ACC CT catalytic site suggest implications for a different binding in the mutant form (V2083) when compared to the wild-type form (A2083) (Figure 5). In the wildtype structure, both compounds fit nicely into the binding pocket with the distances between the Cβ atom of A2083 and C-2 in both ketoenol molecules and the *para*-methyl in spiromesifen between 3.4 Å and 3.9 Å, respectively. However, due to the larger side chain of valine in the resistant ACC variant, the distances between the C_G1_ of the valine and the above-mentioned atoms decrease below 2.8Å, which causes a steric van der Waals clash large enough to impede binding to the resistant mutant. To avoid these intermolecular clashes, the aromatic ring can rotate and shift to a certain extent while still maintaining the overall binding mode. By doing this, the van der Waals clash between the C-2 and the valine can be significantly reduced for spirotetramat (lack of *para*-methyl), while the clash between the *para*-methyl of spiromesifen and mutant valine remains, since rotation of the aromatic ring does not change its position and shifting is not possible due to other close contacts such as Y1819 and V2084 (Figure 5). As a result, resistance ratios based on the A2083V mutation are likely to be higher for spiromesifen when compared to spirotetramat, as shown in bioassays for the non-selected *A. proletella* strains 5/19 and 6/20.

## 4. Discussion

Our study for the first time revealed significant levels of resistance against cyclic ketoenol insecticides in field-collected populations of *A. proletella*. The calculated LC_50_-values of >200 mg L^−1^ for spiromesifen and spirotetramat tested against cabbage whitefly nymphs exceed the recommended label rates for cyclic ketoenols such as spirotetramat in Brassica crops (75 g ha^−1^, 300–600 L ha^−1^). Similar levels of ketoenol resistance have been previously described in cotton whiteflies, *B. tabaci*, known as a notorious pest of greenhouse and field vegetables in Europe [31,35], which evolved resistance to almost all chemical classes of insecticides applied for its control [37]. Ketoenol resistance is not restricted to European *B. tabaci* populations but has recently also been demonstrated in Australian populations [32,34]. Another study reported a shift to lower ketoenol susceptibility in Greek and Spanish populations of greenhouse whiteflies, *T. vaporariorum*, but LC_50_-values for spiromesifen—despite some variation—remained well below the recommended label rates for spiromesifen [36]. Whereas resistance to many chemical classes of insecticides in *B. tabaci* is a well-known phenomenon [37], including the more recent evolution of ketoenol resistance in some regions, only a single case of field-relevant resistance (to pyrethroids) has been reported yet for *A. proletella* [40]; however, the molecular mechanisms conferring pyrethroid resistance in *A. proletella* have not been unveiled yet. Interestingly, we did not find large variation in LC_50_-values for the pyrethroid *lambda*-cyhalothrin and the neonicotinoid acetamiprid against *A. proletella* populations collected in 2019 and 2020, suggesting that both insecticides are principally candidates to manage ketoenol resistance.

Insecticidal ketoenols inhibit ACC by interfering with the CT partial reaction as demonstrated in saturation kinetic experiments by an increase in the apparent *K*_m_ values for acetyl-CoA in the presence of increasing concentrations of spirotetramat-enol, the activated/hydrolyzed form of spirotetramat [29]. It is the same mode of action addressed by ACC inhibiting herbicides, where the evolution of target-site resistance is well documented in certain weed species [49]. A recent study conducted by Bielza et al. [31] suggested a target-site resistance mechanism possibly involved in ketoenol resistance in *B. tabaci*. This assumption was based on the lack of synergism by detoxification enzyme inhibitors such as piperonyl butoxide in bioassays with whitefly field strains expressing high levels of ketoenol resistance. Indeed, it was shown that ketoenol cross-resistance in Spanish and Australian strains of *B. tabaci* is conferred by an ACC target-site mutation, A2083V, in the CT domain of the enzyme. ACC sequence alignments revealed that the mutation is present in a highly conserved region of the enzyme with an alanine at the same position across a broad phylogenetic range of invertebrates such as nematode, spider mite and insect species of different orders, including whiteflies [32].

Here we confirmed by RNAseq analysis the presence of a non-synonymous mutation in the CT domain of assembled ACC variants of various *A. proletella* strains leading to a substitution of an alanine residue by valine at position 2084, which is equivalent to the A2083V mutation described in *B. tabaci*. Selection of partially resistant field strains of *A. proletella* with spiromesifen resulted in strains highly resistant to ketoenols (e.g., SPI-5/19) and supported the importance of the A2083V mutation, as the mutation was almost fixed after two consecutive selection cycles (Table 4). This mutation has been recently functionally validated by CRISPR/Cas9-mediated reverse genetic approaches in mutant *D. melanogaster* [32] and *C. elegans* [33] lines expressing high levels of ketoenol resistance when compared to wild-type lines. Based on these previous findings we conclude that the observed resistance to ketoenols in *A. proletella* is conferred by the same mutation as previously described in *B. tabaci* [32]. This view is supported by molecular modelling studies presented in this study, showing that ketoenol docking is compromised by intermolecular clashes between valine and the aromatic ring substituents such as the mesitylene residue in spiromesifen. Studies with recombinantly expressed mutant and wildtype ACC would provide further insights into ketoenol binding and the importance of other amino acid residues present in the catalytic site; however, it will not change the functional validation of the A2083V target-site mechanism already confirmed by reverse genetics approaches. The importance of this mutation in other pests targeted by ketoenols has been recently highlighted in a clonal culture of *M. persicae* (green peach aphid) highly resistant to spirotetramat and collected in Queensland, Australia [50]. The authors employed a candidate gene approach to unveil the molecular basis of spirotetramat resistance in this aphid clone by mapping RNAseq reads to the ACC gene and identified a A2226V mutation (equivalent to A2083V and A2084V in *B. tabaci* and *A. proletella*, respectively). A subsequent study in Australia revealed the presence of the ketoenol resistance allele (A2226V) in multiple field-collected strains of *M. persicae* [51].

There have been other mutations described in ACC in a few invertebrate pests such as A1079T in *Tetranychus urticae* [38], E645K in *T. vaporariorum* [30,36], and P2170S amongst others in *Aphis gossypii* [52], but these were outside the highly conserved ketoenol binding site in the CT domain and their functional validation either failed or is still pending. Interestingly, a recent phylogenetic study including transcriptomic sequence assemblies and looking for acaricide target-site mutations in Phytoseiid mites revealed the presence of the A2083V mutation in the ACC orthologue of *Amblyseius swirskii* [53], a predatory mite used as a biological control agent for pest management in greenhouse vegetables and ornamentals [54]. It is tempting to speculate that the use of *A. swirskii* in combination with ketoenols in integrated pest management programs might have contributed to the evolution of the resistance trait in *A. swirskii*. Such a powerful biological control agent against whiteflies, thrips, and spider mites with “in-built” ketoenol resistance could be combined with ketoenol insecticides and concurrently applied without losing its efficacy due to acaricidal side effects some insecticidal ketoenols may have.

**Figure 6:**
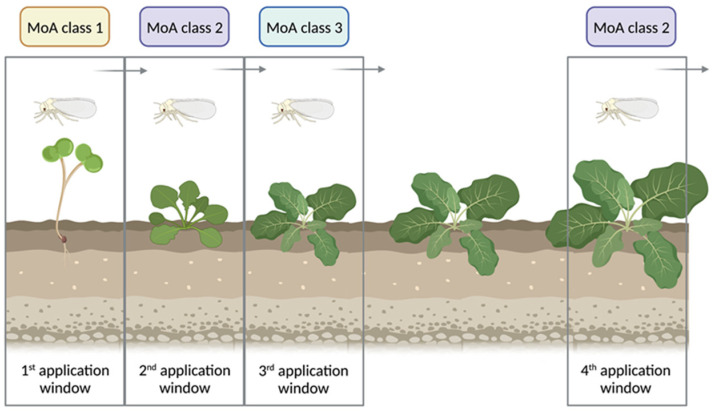
Mode of action (MoA) treatment windows approach for resistance management. Insecticide treatment windows based on different MoA classes aim to manage whitefly populations using the minimum duration of a single generation (e.g., 20–30 days). Multiple applications of the same MoA are possible within a treatment window. When a treatment window is completed, a different MoA class is used in the next treatment window, and, if possible, a different MoA should be applied in a third MoA treatment window. The example shown is based on a situation with three different MoA classes available that work equally well against *Aleyrodes proletella*. (Created with BioRender.com, accessed on 30 October 2022).

An important aspect when considering the implementation of resistance management strategies is the knowledge about the spread of the problem based on resistance allele frequency and how resistance is inherited. Whiteflies such as *A. proletella* are haplo-diploid and males result from unfertilized eggs, i.e., haploid males are either susceptible (ACC wildtype variant) or resistant (ACC A2083V mutant variant), and it has been shown by modelling approaches that resistance allele frequency under certain conditions is increasing faster in haplo-diploid organisms [55]. Ketoenol resistance is an autosomal dominant trait in *A. proletella*; i.e., heterozygous females express a completely resistant phenotype, as revealed by discriminating dose bioassays with F1 progeny from reciprocal crosses showing high survival rates (>94%) comparable to homozygotes. The level of dominance calculated in the present study for *A. proletella* is comparable to results obtained for *B. tabaci* where ketoenol resistance was shown to be autosomal dominant, too [32]. We employed a pyrosequencing assay and screened field-collected samples from several European countries and demonstrated the presence of ketoenol resistance alleles at various frequencies. In some countries such as the Netherlands and Germany, the resistance allele frequency in cabbage whiteflies was quite high, albeit regionally restricted, whereas it was relatively low in others such as Spain. Nevertheless, we strongly recommend the implementation of appropriate resistance management strategies based on a mode of action (MoA) treatment windows approach (Figure 6) as advocated by the Insecticide Resistance Action Committee (IRAC) and previously suggested for cotton whitefly resistance management [37].

In conclusion, based on the findings of the regional genotyping initiative presented here and considering the tendency of increasing global importance of cabbage whitefly infestations, a reinforcement of resistance monitoring studies is warranted covering the most frequently used insecticides such as ketoenols to detect early signs of resistance evolution and spread in Brassica crops. Such an approach will support the implementation of successful cabbage whitefly control measures and sustainable crop yields.

## Figures and Tables

**Table 1:** Field-collected populations of *Aleyrodes proletella*. Selected strains, SPI-5/19, SPI-2/20, and SPI-6/20 were maintained on cabbage plants treated with spiromesifen (200 mg L^−1^).

STRAIN	YEAR	COUNTRY	VENUE	HOST PLANT
1/19	2019	France	Richebourg	Cauliflower
3/19	2019	Croatia	Varazdin	Green cabbage
4/19	2019	France	Warrem	White cabbage
5/19	2019	Belgium	Borgworm	White cabbage
SPI-5/19				Selected 5/19
6/19	2019	Belgium	Lier	White cabbage
1/20	2020	Germany	Helse	White cabbage
2/20	2020	Germany	Blomberg	Green cabbage
SPI-2/20				Selected 2/20
4/20	2020	Germany	Bardowick	White cabbage
5/20	2020	Germany	Bardowick	White cabbage
6/20	2020	Germany	Hannover	Green cabbage
SPI-6/20				Selected 6/20

**Table 2:** Log-dose probit-mortality data for spiromesifen and spirotetramat against 2nd-instar nymphs of field-collected strains of *Aleyrodes proletella.* The resistance ratio (RR) was calculated by dividing the LC_50_ value of the respective field strain by the LC_50_ value of the most susceptible strain (marked with an asterisk *) against spiromesifen and spirotetramat, respectively.

Insecticide	Strain	LC_50_ [mg/L]	95% CI ^a^	Slope ± SE	RR
Spiromesifen	1/19	3.96	3.39–4.63	3.42 ± 0.33	1
	3/19 *	2.76	1.71–4.49	1.87 ± 0.15	1
	4/19	4.33	1.74–11.6	2.29 ± 0.19	2
	5/19	154	45.4–1235	0.9 ± 0.07	56
	SPI-5/19	>200	-	-	>72
	6/19	5.7	3.42–9.33	2.1 ± 0.17	2
	1/20	13.8	8.88–21.3	2.24 ± 0.2	5
	2/20	>200	-	-	>72
	SPI-2/20	>200	-	-	>72
	4/20	9.85	5.23–20.8	2.82 ± 0.26	4
	5/20	4.13	2.34–7.42	1.78 ± 0.14	1
	6/20	>200	-	-	>72
	SPI-6/20	>200	-	-	>72
Spirotetramat	1/19	11.3	6.36–20.9	2.42 ± 0.21	3
	3/19	6.95	5.92–8.17	3.18 ± 0.34	2
	4/19	6.15	4.53–8.41	2.66 ± 0.25	2
	5/19	57.8	37.2–91	1.57 ± 0.12	16
	SPI-5/19	>200	-	-	>56
	6/19	4.24	1.98–9.43	2.48 ± 0.22	1
	1/20 *	3.58	3.08–4.16	3.67 ± 0.34	1
	2/20	>200	-	-	>56
	SPI-2/20	>200	-	-	>56
	4/20	3.7	1.69–7.79	3.26 ± 0.3	1
	5/20	3.63	1.7–8.17	2.55 ± 0.22	1
	6/20	123	103–146	2.76 ± 0.26	34
	SPI-6/20	>200	-	-	>56

^a^ 95% confidence intervals.

**Table 3:** Log-dose probit-mortality data for acetamiprid and λ-cyhalothrin against one-week-old adults of selected strains of *Aleyrodes proletella* in a leaf-dip assay (72 h). The resistance ratio (RR) was calculated by dividing the LC_50_ value of the respective field strain by the LC_50_ value of the most susceptible strain (4/19, marked with an asterisk *) against acetamiprid and λ-cyhalothrin, respectively.

Insecticide	Strain	LC_50_ [mg L^−1^]	95% CI ^a^	Slope ± SE	RR
Acetamiprid	3/19	40.5	7.81–255	1.21 ± 0.08	1
	4/19 *	37.8	8.03–244	1.1 ± 0.07	1
	5/19	75.1	61.3–92.2	1.93 ± 0.15	2
	SPI-5/19	42.1	35.8–49.5	3.09 ± 0.3	1
	6/19	99.6	47.8–220	1.72 ± 0.13	3
	6/20	60.7	31.6–123	1.86 ± 0.14	2
	SPI-6/20	61.7	29.9–135	1.90 ± 0.15	2
λ-cyhalothrin	3/19	52.7	12.6–299	1.29 ± 0.08	2
	4/19 *	27.9	3.59–387	1.02 ± 0.06	1
	5/19	137	95.1–195	2.1 ± 0.18	5
	SPI-5/19	223	100–460	2.24 ± 0.18	8
	6/19	174	142–212	1.98 ± 0.16	6
	6/20	94.8	40.1–245	1.2 ± 0.08	3
	SPI-6/20	74.7	52.2–108	1.73 ± 0.13	3

^a^ 95% confidence intervals.

**Table 4:** Genotyping by pyrosequencing of individuals of different field-collected and laboratory-selected strains of *Aleyrodes proletella* for the presence of the A2083V mutation in the acetyl-CoA carboxylase (ACC) carboxyltransferase (CT) domain. A/A, wildtype homozygote (susceptible); A/V, resistant heterozygote; V/V, resistant homozygote.

	ACC Genotype (%)		
Strain	A/A	A/V	V/V
1/19	100	0	0
3/19	100	0	0
4/19	100	0	0
5/19	70	20	10
SPI-5/19	0	20	80
6/19	100	0	0
1/20	100	0	0
2/20	10	60	30
SPI-2/20	0	10	90
4/20	100	0	0
5/20	100	0	0
6/20	30	0	70
SPI-6/20	0	0	100

## Data Availability

All data generated or analyzed during this study are included in this published article. *A. proletella* transcriptomic data have been submitted to GenBank (TSA: *Aleyrodes proletella*, transcriptome shotgun assembly—Nucleotide—NCBI (nih.gov)) and is available under GJYF01000000, BioProject: PRJNA832135 including Bio Samples: SAMN27777462, SAMN27777463, SAMN27777464, SAMN27777465, SAMN27777466, SAMN27777467.

## Supplementary Materials

The following supporting information can be downloaded at: https://www.mdpi.com/article/10.3390/insects15030178/s1, Table S1: Samples of *Aleyrodes proletella* adults collected in different European countries and preserved in ethanol (70% *v*/*v*) for pyrosequencing diagnostics; Table S2: Primer pairs used for RT-qPCR analysis of *Aleyrodes proletella* acetyl-CoA carboxylase (ACC) expression levels; Table S3: Primer pairs used for *Aleyrodes proletella* acetyl-CoA carboxylase (ACC) genotyping by pyrosequencing; Table S4: Genotyping by pyrosequencing of alcohol-preserved individuals of *Aleyrodes proletella* for the presence of the A2083V mutation in the ACC carboxyltransferase (CT) domain. Genotypes: A/A, susceptible homozygotes; A/V, heterozygotes; V/V, resistant homozygotes; Figure S1: Partial nucleotide sequence of a cDNA fragment of the carboxyltransferase (CT) domain of *Aleyrodes proletella* ACC harboring the mutation site A2083 (red). Annealing positions for primers for pyrosequencing diagnostics of the mutation site A2083V are indicated by blue arrows; Figure S2: Translation of GenBank Acc. No. GJYF01046828.1 representing the amino acid sequence of ACC of *Aleyrodes proletella* strain 3/19. The protein sequences of ACC in strains 3/19, 4/19, 5/20, and 6/19 are identical. Strains 2/20 and SPI-2/20 have A2084V (shown in red); Figure S3: Multiple alignment of ACCase of *Bemisia tabaci*, *Myzus persicae*, predicted ACCase amino acid sequence of *Trialeurodes vaporariorum* and of five strains of *Aleyrodes proletella*, 2/20 (GenBank accession no. GJYF01020973.1), 3/19 (GJYF01046828.1), 4/19 (GJYF01092380.1), 5/20 (GJYF01105771.1) and 6/19 (GJYF01137039.1). The carboxyltransferase (CT) domain (PF01039.35) is highlighted in yellow (AA 1658-2210). Previously described mutations E645K (*T. vaporariorum*) and A2083V (*B. tabaci*) are highlighted in blue.

## References

[1] Martin J.H., Mifsud D., Rapisarda C. (2000). The Whiteflies (Hemiptera: Aleyrodidae) of Europe and the Mediterranean Basin. Bull. Entomol. Res..

[2] Martin J.H., Mound L.A. (2007). An Annotated Check List of the World’s Whiteflies (Insecta: Hemiptera: Aleyrodidae). Zootaxa.

[3] Chen M.-M., Guo R., Zhang J.-L., Wan G.-H., Yang J., Zhang G.-F. (2015). Rapid Identification of *Aleyrodes proletella* (Hemiptera: Aleyrodidae), a New Invasive Whitefly Species in Mainland China, Based on SS-COI Marker. Acta Entomol. Sin..

[4] Zhang G.F., Xian X.Q., Zhang J.L., Li X.F., Ma D.Y., Wan F.H. (2014). Cabbage Whitefly, *Aleyrodes proletella* (L.) (Hemiptera: Aleyrodidae), Invaded Mainland China. Entomol. J. East China.

[5] Alexander Jesudasan R.W., David B.V. (1991). Taxonomic Studies on Indian Aleyrodidae (Insecta: Homoptera). Orient. Insects.

[6] De Barro P.J., Carver M. (1997). Cabbage Whitefly, *Aleyrodes proletella* (L.) (Hemiptera: Aleyrodidae), Newly Discovered in Australia. Aust. J. Entomol..

[7] Trdan S., Papler U. (2002). Susceptibility of Four Different Vegetable Brassicas to Cabbage Whitefly (*Aleyrodes proletella* L., Aleyrodidae) Attack. Meded. Rijksuniv. Te Gent Fak. Van Landbouwkd. En Toegepaste Biol. Wet..

[8] Richter E., Hirthe G. (2014). Hibernation and Migration of *Aleyrodes proletella* in Germany. IOBC/WPRS Bull..

[9] (2019). OEPP/EPPO PP 1/311 (1) *Aleyrodes proletella* on Brassica Crops. EPPO Bull..

[10] Saucke H., Schultz B., Wedemeyer R., Liebig N., Zimmermann O., Katz P. (2011). Biotechnische Regulierung der Kohlmottenschildlaus in Kohlgemüse—Sachstand und Perspektiven. Gesunde Pflanz..

[11] Carden P.W. (1972). New or Uncommon Plant Diseases and Pests. Plant Pathol..

[12] Alonso D., Gómez A.A., Nombela G., Muñiz M. (2009). Temperature-Dependent Development of *Aleyrodes proletella* (Homoptera: Aleyrodidae) on Two Cultivars of Broccoli under Constant Temperatures. Environ. Entomol..

[13] Butler C.G. (1938). On the Ecology of *Aleurodes brassicae* Walk. (Hemiptera). Trans. R. Entomol. Soc. Lond..

[14] Pajović I. (2011). Seasonal Dynamics of Most Detrimental Pest Insects Species on Cabbage Plants in Montenegro. Agric. For..

[15] Kovaříková K., Holý K., Skuhrovec J., Saska P. (2017). The Efficacy of Insecticides against Eggs and Nymphs of Aleyrodes Proletella (Hemiptera: Aleyrodidae) under Laboratory Conditions. Crop Prot..

[16] Laurenz S., Schmidt S., Balkenhol B., Meyhofer R. (2019). Natural Enemies Associated with the Cabbage Whitefly *Aleyrodes proletella* in Germany. J. Plant Dis. Prot..

[17] Koca A.S., Kütük H. (2020). Distribution, Host Plants and Natural Enemies of *Aleyrodes proletella* L. (Hemiptera: Aleyrodidae) on Collard (*Brassica oleracea* L. Var. Acephala) in Düzce Province of Turkey. Plant Prot. Bull..

[18] Laurenz S., Meyhöfer R. (2021). Banker Plants Promote Functional Biodiversity and Decrease Populations of the Cabbage Whitefly *Aleyrodes proletella*. J. Appl. Entomol..

[19] Broekgaarden C., Riviere P., Steenhuis G., del sol Cuenca M., Kos M., Vosman B. (2012). Phloem-Specific Resistance in Brassica Oleracea against the Whitefly *Aleyrodes proletella*. Entomol. Exp. Appl..

[20] Hondelmann P., Paul C., Schreiner M., Meyhöfer R. (2020). Importance of Antixenosis and Antibiosis Resistance to the Cabbage Whitefly (*Aleyrodes proletella*) in Brussels Sprout Cultivars. Insects.

[21] Richter E., Hirthe G. (2014). First Results on Population Dynamics and Chemical Control of *Aleyrodes proletella* in Germany. IOBC-WPRS Bull..

[22] Bretschneider T., Benet-Buchholz J., Fischer R., Nauen R. (2003). Spirodiclofen and Spiromesifen—Novel Acaricidal and Insecticidal Tetronic Acid Derivatives with a New Mode of Action. Chimia.

[23] Marcic D., Peric P., Petronijevic S., Prijovic M., Drobnjakovic T. (2011). Cyclic Ketoenols: Acaricides and Insecticides with a Novel Mode of Action. Pestic. Fitomed..

[24] Brück E., Elbert A., Fischer R., Krueger S., Kühnhold J., Klueken A.M., Nauen R., Niebes J.-F., Reckmann U., Schnorbach H.-J. (2009). Movento^®^, an Innovative Ambimobile Insecticide for Sucking Insect Pest Control in Agriculture: Biological Profile and Field Performance. Crop Prot..

[25] Nauen R., Konanz S. (2005). Spiromesifen as a New Chemical Option for Resistance Management in Whiteflies and Spider Mites. Bayer Crop. J..

[26] Nauen R., Reckmann U., Thonzik J., Thielert W. (2008). Biological Profile of Spirotetramat (Movento)—A New Two-Way Systemic (Ambimobile) Insecticide against Sucking Pest Species. Bayer Crop. J..

[27] Muehlebach M., Buchholz A., Zambach W., Schaetzer J., Daniels M., Hueter O., Kloer D.P., Lind R., Maienfisch P., Pierce A. (2020). Spiro N-Methoxy Piperidine Ring Containing Aryldiones for the Control of Sucking Insects and Mites: Discovery of Spiropidion. Pest Manag. Sci..

[28] Parvy J.-P., Napal L., Rubin T., Poidevin M., Perrin L., Wicker-Thomas C., Montagne J. (2012). Drosophila Melanogaster Acetyl-CoA-Carboxylase Sustains a Fatty Acid-Dependent Remote Signal to Waterproof the Respiratory System. PLoS Genet..

[29] Lümmen P., Khajehali J., Luther K., Van Leeuwen T. (2014). The Cyclic Keto-Enol Insecticide Spirotetramat Inhibits Insect and Spider Mite Acetyl-CoA Carboxylases by Interfering with the Carboxyltransferase Partial Reaction. Insect Biochem. Mol. Biol..

[30] Karatolos N., Williamson M.S., Denholm I., Gorman K., Ffrench-Constant R., Nauen R. (2012). Resistance to Spiromesifen in *Trialeurodes vaporariorum* Is Associated with a Single Amino Acid Replacement in Its Target Enzyme Acetyl-Coenzyme A Carboxylase. Insect Mol. Biol..

[31] Bielza P., Moreno I., Belando A., Grávalos C., Izquierdo J., Nauen R. (2019). Spiromesifen and Spirotetramat Resistance in Field Populations of *Bemisia tabaci* Gennadius in Spain. Pest Manag. Sci..

[32] Lueke B., Douris V., Hopkinson J.E., Maiwald F., Hertlein G., Papapostolou K.-M., Bielza P., Tsagkarakou A., Van Leeuwen T., Bass C. (2020). Identification and Functional Characterization of a Novel Acetyl-CoA Carboxylase Mutation Associated with Ketoenol Resistance in *Bemisia tabaci*. Pestic. Biochem. Physiol..

[33] Guest M., Kriek N., Flemming A.J. (2020). Studies of an Insecticidal Inhibitor of Acetyl-CoA Carboxylase in the Nematode C. Elegans. Pestic. Biochem. Physiol..

[34] Hopkinson J., Balzer J., Fang C., Walsh T. (2023). Insecticide Resistance Management of *Bemisia tabaci* (Hemiptera: Aleyrodidae) in Australian Cotton—Pyriproxyfen, Spirotetramat and Buprofezin. Pest Manag. Sci..

[35] Mavridis K., Papapostolou K.M., Ilias A., Michaelidou K., Stavrakaki M., Roditakis E., Tsagkarakou A., Bass C., Vontas J. (2022). Next-Generation Molecular Diagnostics (TaqMan qPCR and ddPCR) for Monitoring Insecticide Resistance in *Bemisia tabaci*. Pest Manag. Sci..

[36] Kapantaidaki D.E., Sadikoglou E., Tsakireli D., Kampanis V., Stavrakaki M., Schorn C., Ilias A., Riga M., Tsiamis G., Nauen R. (2018). Insecticide Resistance in *Trialeurodes vaporariorum* Populations and Novel Diagnostics for Kdr Mutations. Pest Manag. Sci..

[37] Horowitz A.R., Ghanim M., Roditakis E., Nauen R., Ishaaya I. (2020). Insecticide Resistance and Its Management in *Bemisia tabaci* Species. J. Pest Sci..

[38] De Rouck S., İnak E., Dermauw W., Van Leeuwen T. (2023). A Review of the Molecular Mechanisms of Acaricide Resistance in Mites and Ticks. Insect Biochem. Mol. Biol..

[39] İnak E., Demirci B., Vandenhole M., Söylemezoğlu G., Van Leeuwen T., Toprak U. (2023). Molecular Mechanisms of Resistance to Spirodiclofen and Spiromesifen in *Tetranychus urticae*. Crop Prot..

[40] Springate S., Colvin J. (2012). Pyrethroid Insecticide Resistance in British Populations of the Cabbage Whitefly, *Aleyrodes proletella*. Pest Manag. Sci..

[41] Horowitz A.R., Gorman K., Ross G., Denholm I. (2003). Inheritance of Pyriproxyfen Resistance in the Whitefly, *Bemisia tabaci* (Q Biotype). Arch. Insect Biochem. Physiol..

[42] Liu Y., Tabashnik B.E. (1997). Inheritance of Resistance to the Bacillus Thuringiensis Toxin Cry1C in the Diamondback Moth. Appl. Environ. Microbiol..

[43] Grabherr M.G., Haas B.J., Yassour M., Levin J.Z., Thompson D.A., Amit I., Adiconis X., Fan L., Raychowdhury R., Zeng Q. (2011). Full-Length Transcriptome Assembly from RNA-Seq Data without a Reference Genome. Nat. Biotechnol..

[44] Wu T.D., Watanabe C.K. (2005). GMAP: A Genomic Mapping and Alignment Program for mRNA and EST Sequences. Bioinformatics.

[45] Sievers F., Wilm A., Dineen D., Gibson T.J., Karplus K., Li W., Lopez R., McWilliam H., Remmert M., Söding J. (2011). Fast, Scalable Generation of High-Quality Protein Multiple Sequence Alignments Using Clustal Omega. Mol. Syst. Biol..

[46] Yu L.P.C., Kim Y.S., Tong L. (2010). Mechanism for the Inhibition of the Carboxyltransferase Domain of Acetyl-Coenzyme A Carboxylase by Pinoxaden. Proc. Natl. Acad. Sci. USA.

[47] Schneider N., Lange G., Hindle S., Klein R., Rarey M. (2013). A Consistent Description of HYdrogen Bond and DEhydration Energies in Protein-Ligand Complexes: Methods behind the HYDE Scoring Function. J. Comput. Aided Mol. Des..

[48] Abbott W.S. (1925). A Method of Computing the Effectiveness of an Insecticide. J. Econ. Entomol..

[49] Kaundun S.S. (2014). Resistance to Acetyl-CoA Carboxylase-Inhibiting Herbicides. Pest Manag. Sci..

[50] Singh K.S., Cordeiro E.M.G., Troczka B.J., Pym A., Mackisack J., Mathers T.C., Duarte A., Legeai F., Robin S., Bielza P. (2021). Global Patterns in Genomic Diversity Underpinning the Evolution of Insecticide Resistance in the Aphid Crop Pest *Myzus persicae*. Commun. Biol..

[51] Umina P.A., Bass C., van Rooyen A., Chirgwin E., Arthur A.L., Pym A., Mackisack J., Mathews A., Kirkland L. (2022). Spirotetramat Resistance in *Myzus persicae* (Sulzer) (Hemiptera: Aphididae) and Its Association with the Presence of the A2666V Mutation. Pest Manag. Sci..

[52] Pan Y., Zhu E., Gao X., Nauen R., Xi J., Peng T., Wei X., Zheng C., Shang Q. (2017). Novel Mutations and Expression Changes of Acetyl-Coenzyme A Carboxylase Are Associated with Spirotetramat Resistance in *Aphis gossypii* Glover: Overexpression of Mutated ACC Account for Spirotetramat Resistance. Insect Mol. Biol..

[53] Bajda S.A., De Clercq P., Van Leeuwen T. (2022). Selectivity and Molecular Stress Responses to Classical and Botanical Acaricides in the Predatory Mite Phytoseiulus Persimilis Athias-Henriot (Acari: Phytoseiidae). Pest Manag. Sci..

[54] Buitenhuis R., Murphy G., Shipp L., Scott-Dupree C. (2015). *Amblyseius swirskii* in Greenhouse Production Systems: A Floricultural Perspective. Exp. Appl. Acarol..

[55] Helps J.C., Paveley N.D., van den Bosch F. (2017). Identifying Circumstances under Which High Insecticide Dose Increases or Decreases Resistance Selection. J. Theor. Biol..

