# Supplementary material for: Mapping and Characterization of Target-Site Resistance to Cyclic Ketoenol Insecticides in Cabbage Whiteflies, Aleyrodes proletella (Hemiptera: Aleyrodidae)
Source: Insects. 2024 Mar 6;15(3):178. doi: 10.3390/insects15030178 (PMC10970768; doi:10.3390/insects15030178)
Supplement: Supplementary file 1 [file insects-15-00178-s001.zip › insects-2897919-supplementary.pdf]

SUPPLEMENT

Mapping and characterization of target-site resistance to cyclic ketoenol insecticides in cabbage whiteflies, *Aleyrodes proletella* (Hemiptera: Aleyrodidae)

Viola Müller[1,2], Frank Maiwald[1], Gudrun Lange[3] and Ralf Nauen[1,*]

[1] Bayer AG, Crop Science Division, R&D, 40789 Monheim, Germany
[2] University of Bonn, INRES, 53115 Bonn, Germany
[3] Bayer AG, Crop Science Division, R&D, 65926 Frankfurt, Germany
* Correspondence: ralf.nauen@bayer.com; Tel.:+49-2173-384441

Table S1. Samples of *Aleyrodes proletella* adults collected in different European countries and preserved in ethanol (70% v/v) for pyrosequencing diagnostic.

Year	Sample-ID	Country	Venue	Host
**2019**	NAR2019-131	Netherlands	Mijnsherenland	Brussels sprouts
	NAR2019-132	Netherlands	Klaaswaal	Brussels sprouts
	NAR2019-148	Poland	Warsaw	Unknown
	NAR2019-149	Poland	Warsaw	Unknown
	NAR2019-150	Netherlands	Schagerbrug	Green cabbage
	NAR2019-151	Netherlands	Oosternijkerk	Broccoli
	NAR2019-162	Switzerland	Zollikofen	Brussels sprouts
	NAR2019-163	Spain	Andalucia	Broccoli
	NAR2019-164	Spain	Murcia	Broccoli
	NAR2019-165	Spain	Murcia	Broccoli
	NAR2019-166	Spain	Murcia	Broccoli
	NAR2019-183	Spain	La Rioja	Cauliflower
**2020**	NAR2020-119	Belgium	Roeselare	Brussels sprouts
	NAR2020-120	Belgium	Lier	Brussels sprouts
	NAR2020-121	Belgium	Kruishoutem (Kruisem)	Brussels sprouts
	NAR2020-122	Netherlands	Den Bommel	Brussels sprouts
	NAR2020-123	Netherlands	Oud-Beijerland	Brussels sprouts
	NAR2020-124	Netherlands	Heerjansdam	Brussels sprouts
**2021**	NAR2021-048	Spain	Picassent	Cabbage
	NAR2021-049	Spain	Picassent	Cabbage
	NAR2021-050	Spain	Alzira	Cabbage
	NAR2021-051	Spain	Simat de la Valldigna	Cabbage
	NAR2021-052	Spain	Algemesi	Cabbage
	NAR2021-053	Spain	Alzira	Cabbage
	NAR2021-054	Spain	Manuel	Cabbage
	NAR2021-055	Spain	Simat de la Valldigna	Cabbage
	NAR2021-060	Spain	Finca Las Cabecicas Lorca	Cabbage
	NAR2021-061	Spain	Lorca	Cabbage
	NAR2021-110	Netherlands	Den Bommel	Brussel sprouts
	NAR2021-111	Netherlands	Hellevoetsluis	Brussel sprouts
	NAR2021-112	Netherlands	Mijnsheerenland	Brussel sprouts
	NAR2021-113	Netherlands	Bruinisse	Cabbage
	NAR2021-114	Netherlands	Zeewolde	Cabbage
	NAR2021-115	Netherlands	Den Bommel	Cabbage
	NAR2021-116	Netherlands	Hellvoetsluis	Cabbage
	NAR2021-117	Netherlands	Mijnsheerenland	Cabbage
	NAR2021-120	Netherlands	Sexbierum	Cabbage
	ALTHFL_DEU21_0001	Germany	Hannover	Savoy cabbage
	ALEUPR_DEU21_0002	Germany	Hannover	Savoy cabbage

Table S2. Primer pairs used for RT-qPCR analysis of *Aleyrodes proletella* acetyl-CoA carboxylase (ACC) expression levels.

Primer	Forward primer (5' to 3')	Reverse primer (5' to 3')	Amplicon, bp
ACC	AGGTACGGCGCCAGATTATG	AACCACTGTCGTTCGCTAGG	121
Actin	CGGTCGTACCACTGGTATCG	AAACGGAGGATGGCATGAGG	99
ATP	AAGTCAACCACCGAGTCAGC	TGAAGACCTTTCTGCCGAGC	129
HSP-90	AGCTCTCCGTGCTAAAGCTG	CTGATCATGCGGTGAATGCG	147

Table S3. Primer pairs used for *Aleyrodes proletella* acetyl-CoA carboxylase (ACC) genotyping by pyrosequencing.

Primer	Forward primer (5' to 3')	Reverse primer (5' to 3')	Amplicon, bp
ACC A2083V	TGTGGATGAGTTACGTGTTTACAA	[btn]GGGGTTAATGGTGGGATCAACTA	
ACC A2083V_seq	AGAGGAGGAGCTTGG		106

Table S4. Genotyping by pyrosequencing of alcohol-preserved individuals of *Aleyrodes proletella* for the presence of the A2083V mutation in the ACC carboxyltransferase (CT) domain. Genotypes: A/A, susceptible homozygotes; A/V, heterozygotes; V/V, resistant homozygotes.

			Genotype (%)		
**Sample**	**Country**	**Year**	**A/A**	**A/V**	**V/V**
NAR2019-131	Netherlands	2019	100	0	0
NAR2019-132	Netherlands	2019	100	0	0
NAR2019-148	Poland	2019	100	0	0
NAR2019-149	Poland	2019	90	10	0
NAR2019-150	Netherlands	2019	100	0	0
NAR2019-151	Netherlands	2019	100	0	0
NAR2019-162	Switzerland	2019	0	0	100
NAR2019-163	Spain	2019	100	0	0
NAR2019-164	Spain	2019	100	0	0
NAR2019-165	Spain	2019	100	0	0
NAR2019-166	Spain	2019	100	0	0
NAR2019-183	Spain	2019	100	0	0
NAR2020-119	Belgium	2020	100	0	0
NAR2020-120	Belgium	2020	100	0	0
NAR2020-121	Belgium	2020	100	0	0
NAR2020-122	Netherlands	2020	0	0	100
NAR2020-123	Netherlands	2020	0	30	70
NAR2020-124	Netherlands	2020	0	50	50
NAR2021-048	Spain	2021	100	0	0
NAR2021-049	Spain	2021	100	0	0
NAR2021-050	Spain	2021	100	0	0
NAR2021-051	Spain	2021	100	0	0
NAR2021-052	Spain	2021	100	0	0
NAR2021-053	Spain	2021	100	0	0
NAR2021-054	Spain	2021	0	100	0
NAR2021-055	Spain	2021	20	80	0
NAR2021-060	Spain	2021	100	0	0
NAR2021-061	Spain	2021	100	0	0
NAR2021-110	Netherlands	2021	0	10	90
NAR2021-111	Netherlands	2021	0	0	100
NAR2021-112	Netherlands	2021	0	10	90
NAR2021-113	Netherlands	2021	0	10	90
NAR2021-114	Netherlands	2021	100	0	0
NAR2021-115	Netherlands	2021	0	10	90
NAR2021-116	Netherlands	2021	0	0	100
NAR2021-117	Netherlands	2021	0	0	100
NAR2021-120	Netherlands	2021	100	0	0
ALTHFL_DEU21_0001	Germany	2021	30	40	30
ALEUPR_DEU21_0002	Germany	2021	0	60	40

Figure S1. Partial nucleotide sequence of a cDNA fragment of the carboxyltransferase (CT) domain of *Aleyrodes proletella* ACC harboring the mutation site A2083 (red). Annealing positions for primers for pyrosequencing diagnostics of the mutation site A2083V are indicated by blue arrows.

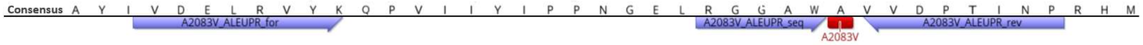

Figure S2. Translation of GenBank Acc. No. GJYF01046828.1 representing the amino acid sequence of ACC of *Aleyrodes proletella* strain 3/19. The protein sequences of ACC in strains 3/19, 4/19, 5/20 and 6/19 are identical. Strains 2/20 and SPI-2/20 have A2084V (shown in red).

```
   1 MEGDTGPKTN NVNFIVGDDG QDGEIANGLI NTISEEDERK ENRDSFPSGN
  51 GPTGLISNSS SYNNMFGLAE RRKRLRPSMS QGTVIHQRLL DKDFTVGTPE
 101 EFVKRFKGTR VINKVLIANN GIAAVKCMRS IRRWSYEMFK NERAVRFVVM
 151 VTPEDLKANA EYIKMADHYV PVPGGTNNNN YANVELIVDI ALRTQVQAVW
 201 AGWGHASENP KLPELLHKNN IAFIGPPEKA MWALGDKIAS SIVAQTAEIP
 251 TLPWSGSELT AQYSGKKIKI SSDLYKKGCV SSVEEGLRSA HKIGFPIMIK
 301 ASEGGGGKGI RKVEAAEEFP NLFRQVQSEV PGSPIFIMKL ARCARHLEVQ
 351 LLADQYGNAI SLFGRDCSIQ RRHQKIIEEA PAVIAEPDVF ENMEKAAVRL
 401 AKMVGYVSAG TVEYLYDPSE GQYFFLELNP RLQVEHPCTE MVADVNLPAA
 451 QLQIAMGLQL NCIKDIRLLY GESPWGDNYI DFDEPRHKPQ PWGHVIAARI
 501 TSENPDEGFK PSSGTVQELN FRSSKNVWGY FSVAASGGLH EFADSQFGHC
 551 FSWGENREQA RENLVIALKE LSIRGDFRTT VEYLITLLET ESFQINTIDT
 601 AWLDLLISER VQSEKPDVLL GVMCGALHIA DRKVCDAFQN FQTSLERGQI
 651 QGCNTLDHNV QVELIHDGLK YKVHATKSGL NSYFLVMNGS FKEIELHRLS
 701 DGGILLSVDG SSFTTYMREE VDRYRIVIGN LTCVFEKEND PSLLRSPSAG
 751 KLISYLIEDG GHVSKGQAYA EIEVMKMVMT LTANESGLVT FAKRPGAVLD
 801 AGSLIGTLEL DDPSLVSKAL DYKGEFPELD VSTPMVGEKL NHAHNHYRQM
 851 LDNILAGFCL PDPYHLVRLR EVIEKFMSSL RDPSLPLLEL QEVISSISGR
 901 IPISVEKKIR KLMTLYERNI TSVLAQFPSQ QIAGVIDSHA ATLQKRSDRD
 951 GFFLTTQGIV QLVQRYRNGI RGRMKSAVHE LLRQYYEVES QFQQGHYDKC
1001 VMAIREKFKD DMAAVTSTIF SHGQVAKKNM LVTMLIDHLW SNEPGLTDEL
1051 ATTLNELTSL NRSEHSRVAL RSRQVLIAAH QPAYELRHNQ MESIFLSAVD
1101 MYGHDFHPEN LQKLIQSETS IFDILHDFFY HTNRAVCNAA LEVYVRRVYI
1151 SYELTCLQHL ELSGEVPLVQ FQFLLPSSHP NRQKVTDPSS PIIADSPETE
1201 EPPSSTPTII HSYQRTGCMA AFESFDQFES YYDEILDLLD ELSPSTISPR
1251 IMEALESGSE SRMSTSINVS LSVGDNPRPP GLEDGLQVEP IHILCIAVKD
1301 NGDLEDEKLS KLLGDFCAKH REELKEKSIR RITFLALNRR QFPKLFTFRN
1351 CDNFVEDRIY RHLEPGMAFQ LELNRMKTYH LEALPTSNRK MYLYLGRAKV
1401 AKGQEVTDFR FFIRSIIRHS DLITKEASFE YLQNEGERVL LEAMDELEVA
1451 FSHPLARRTD CNHIFLNFVP TVIMDSAKIE ESVTNMVMRY GARLWKLRVL
1501 QAELRMTIRT SPNSKTTNVR LCLANDSGYY LDICLYKEVV DPKTGIIKLE
1551 SYGSKQGPLH GLPTAIPYVT KDYLQQKRFQ AQSAGTTYCH DIPDMFRQMV
1601 ERQWKEFIEQ RPNDGIVKPS QLMDYVELVL EDDHLVEQKR LAGENTIGMV
1651 AWRITLNTPE YPDGREIIVI ANDITVRIGS FGPEEDLVFD LASKIARQMK
1701 VPRIYIAANS GARIGLAEEV KSLFRVAWED PDEPDKGLKY LYLSTEDFIK
1751 ISACNSVRAI LIEDEGEARY KITDIIGKED GLGVENLRYA GMIAGETSEA
1801 YREIVTISMV SCRAIGIGAY LVRLGQRVIQ VENSHIILTG YSALNKLLGR
1851 EVYASNNQLG GIQIMYNNGI SHKTEPRDLD GIYSIVKWLG YIPKDKYSPV
1901 PVIKPADPVD REVGYMPTKT PYDPRWMIAG RYSPNNSNEW ESGFFDEGSW
1951 EEVMQPWAQT VVVGRARLGG IPMGVIAVET RTVEVKLPAD PANLDSEAKT
2001 LSQAGQVWFP DSAFKTAQAI KDFEHEDLPL IIFANWRGFS GGMKDMYEQV
2051 MKFGAYIVDE LRVYKQPVII YIPPNGELRG GAWAVVDPTI NPRHMEMYAD
2101 PESRGGVLEP EGIVEIKFRE KDILKTMHRI DQVIVPLKQK LAVPDLSPEE
2151 KAEVENQIVE REQYLKPMYH QVAIHFADLH DTPERMMEKG VIHNIVPWRK
2201 SRTLLHWRVK RLLLENQIKC NLLKVQPQMD NGQAQAMLRR WFVEDKGTTT
2251 AYLWENNESV VAWLMSQLSP DGSISPNSIV ANNIRCVQRD ALINQIKTSM
2301 EESPDVAQDA VVEMFQTLSA NERSEVLRKL SHLETISKPE PQS        2343
```

Figure S3. Multiple alignment of ACCase of *Bemisia tabaci*, *Myzus persicae*, predicted ACCase amino acid sequence of *Trialeurodes vaporariorum* and of five strains of *Aleyrodes proletella*, 2/20 (GenBank accession no. GJYF01020973.1; identical to SPI-2/20), 3/19 (GJYF01046828.1), 4/19 (GJYF01092380.1), 5/20 (GJYF01105771.1) and 6/19 (GJYF01137039.1). The carboxyltransferase (CT) domain (PF01039.35) is highlighted in yellow (AA 1658-2210). Previously described mutations E645K (*T. vaporariorum*; Karatolos et al., 2012) and A2083V (*B. tabaci;* Lueke et al., 2020) are highlighted in blue.

```
                       1                                                  50
BEMITA_QJQ31013        MEGETGQKAN NVNFIVGDDG QDGEIPNGIN T..ISEEDER ERRENRDSFP
MYZUPE_XP_022181497    ~~~MSSETSG GVNFIVGDED GVDQAPAELV NGELMKALEA EKHENTDSFP
TRIAVA_ACCase          ~~~~~~~~~~ ~~~~~~~~~~ ~~~~~~~~~~ ~~~~~~~~~~ ~~~~~~~~~~
2/20 & SPI-2/20        MEGDTGPKTN NVNFIVGDDG QDGEIANGLI N..TI.SEED ERKENRDSFP
3/19                   MEGDTGPKTN NVNFIVGDDG QDGEIANGLI N..TI.SEED ERKENRDSFP
4/19                   MEGDTGPKTN NVNFIVGDDG QDGEIANGLI N..TI.SEED ERKENRDSFP
5/20                   MEGDTGPKTN NVNFIVGDDG QDGEIANGLI N..TI.SEED ERKENRDSFP
6/19                   MEGDTGPKTN NVNFIVGDDG QDGEIANGLI N..TI.SEED ERKENRDSFP

                       51                                                100
BEMITA_QJQ31013        SGNG.PTGII SSSSSYKDMF GLAERRKRLR PSMSQGTVIH QRLLDKDFTV
MYZUPE_XP_022181497    LGKETRMGVT SNSSSYNNMF GLTEKRKRLR PSMSQGNVIH QRLTEKDFNV
TRIAVA_ACCase          ~~~~~~~~~~ ~~~~~~~~~~ ~~~~~~~~~~ ~~MSQGTVIH QRLLDKDFTV
220O                   SGNG.PTGLI SNSSSYNNMF GLAERRKRLR PSMSQGTVIH QRLLDKDFTV
319                    SGNG.PTGLI SNSSSYNNMF GLAERRKRLR PSMSQGTVIH QRLLDKDFTV
419                    SGNG.PTGLI SNSSSYNNMF GLAERRKRLR PSMSQGTVIH QRLLDKDFTV
520                    SGNG.PTGLI SNSSSYNNMF GLAERRKRLR PSMSQGTVIH QRLLDKDFTV
619                    SGNG.PTGLI SNSSSYNNMF GLAERRKRLR PSMSQGTVIH QRLLDKDFTV

                       101                                               150
BEMITA_QJQ31013        GTPEEFVKRF KGTRVINKVL IANNGIAAVK CMRSIRRWSY EMFKNERAVR
MYZUPE_XP_022181497    STPEEFVKRF KGTRVINKVL IANNGIAAVK CMRSVRRWSY EMFRNERAVR
TRIAVA_ACCase          GTPEEFVKRF KGTRVINKVL IANNGIAAVK CMRSIRRWSY EMFKNERAVR
220O                   GTPEEFVKRF KGTRVINKVL IANNGIAAVK CMRSIRRWSY EMFKNERAVR
319                    GTPEEFVKRF KGTRVINKVL IANNGIAAVK CMRSIRRWSY EMFKNERAVR
419                    GTPEEFVKRF KGTRVINKVL IANNGIAAVK CMRSIRRWSY EMFKNERAVR
520                    GTPEEFVKRF KGTRVINKVL IANNGIAAVK CMRSIRRWSY EMFKNERAVR
619                    GTPEEFVKRF KGTRVINKVL IANNGIAAVK CMRSIRRWSY EMFKNERAVR

                       151                                               200
BEMITA_QJQ31013        FVVMVTPEDL KANAEYIKMA DHYVPVPGGT NNNNYANVEL IVDIALRTQV
MYZUPE_XP_022181497    FVVMVTPEDL KANAEYIKMA DHYVPVPGGT NNNNYANVEL IVNIAIRSQV
TRIAVA_ACCase          FVVMVTPEDL KANAEYIKMA DHYVPVPGGT NNNNYANVEL IIDIATRTQV
220O                   FVVMVTPEDL KANAEYIKMA DHYVPVPGGT NNNNYANVEL IVDIALRTQV
319                    FVVMVTPEDL KANAEYIKMA DHYVPVPGGT NNNNYANVEL IVDIALRTQV
419                    FVVMVTPEDL KANAEYIKMA DHYVPVPGGT NNNNYANVEL IVDIALRTQV
520                    FVVMVTPEDL KANAEYIKMA DHYVPVPGGT NNNNYANVEL IVDIALRTQV
619                    FVVMVTPEDL KANAEYIKMA DHYVPVPGGT NNNNYANVEL IVDIALRTQV

                       201                                               250
BEMITA_QJQ31013        QAVWAGWGHA SENPKLPELL HKNNIAFIGP PEKAMWALGD KIASSIVAQT
MYZUPE_XP_022181497    QAVWAGWGHA SENPELPKLL DKNKIAFIGP PEKAMFALGD KIASSIVAQT
TRIAVA_ACCase          QAVWAGWGHA SENPKLPELL HKNNIAFIGP PEKAMWALGD KIASSIVAQT
220O                   QAVWAGWGHA SENPKLPELL HKNNIAFIGP PEKAMWALGD KIASSIVAQT
319                    QAVWAGWGHA SENPKLPELL HKNNIAFIGP PEKAMWALGD KIASSIVAQT
419                    QAVWAGWGHA SENPKLPELL HKNNIAFIGP PEKAMWALGD KIASSIVAQT
520                    QAVWAGWGHA SENPKLPELL HKNNIAFIGP PEKAMWALGD KIASSIVAQT
```

```
619                  QAVWAGWGHA SENPKLPELL HKNNIAFIGP PEKAMWALGD KIASSIVAQT

                     251                                              300
BEMITA_QJQ31013      AEIPTLPWSG SELTAQYSGR KIKISSELYK RGCVSSVEEG LRSAQKIGFP
MYZUPE_XP_022181497  AEIPTLPWSG SGVVGHYSGK KIEIGPDLYK KGCVASIEEG LVSAEKVGYP
TRIAVA_ACCase        AEIPT..... .......... ...ISSELYK KGCVQNVEEG LRSAQKIGFP
220O                 AEIPTLPWSG SELTAQYSGK KIKISSDLYK KGCVSSVEEG LRSAHKIGFP
319                  AEIPTLPWSG SELTAQYSGK KIKISSDLYK KGCVSSVEEG LRSAHKIGFP
419                  AEIPTLPWSG SELTAQYSGK KIKISSDLYK KGCVSSVEEG LRSAHKIGFP
520                  AEIPTLPWSG SELTAQYSGK KIKISSDLYK KGCVSSVEEG LRSAHKIGFP
619                  AEIPTLPWSG SELTAQYSGK KIKISSDLYK KGCVSSVEEG LRSAHKIGFP

                     301                                              350
BEMITA_QJQ31013      VMIKASEGGG GKGIRKVESS EEFPNLFRQV QSEVPGSPIF IMKLARCARH
MYZUPE_XP_022181497  IMIKASEGGG GKGIRKVENT EEFPNAYKQV QAEVPGSPIF IMKLAKCARH
TRIAVA_ACCase        VMVKASEGGG GKGIRKVESS EEFPNLFRQV QAEVPGSPIF IMKLARCARH
220O                 IMIKASEGGG GKGIRKVEAA EEFPNLFRQV QSEVPGSPIF IMKLARCARH
319                  IMIKASEGGG GKGIRKVEAA EEFPNLFRQV QSEVPGSPIF IMKLARCARH
419                  IMIKASEGGG GKGIRKVEAA EEFPNLFRQV QSEVPGSPIF IMKLARCARH
520                  IMIKASEGGG GKGIRKVEAA EEFPNLFRQV QSEVPGSPIF IMKLARCARH
619                  IMIKASEGGG GKGIRKVEAA EEFPNLFRQV QSEVPGSPIF IMKLARCARH

                     351                                              400
BEMITA_QJQ31013      LEVQLLADQY GNAISLFGRD CSIQRRHQKI IEEAPAVIAE PEIFEDMEKA
MYZUPE_XP_022181497  LEVQLLADQY GNAISLFGRD CSIQRRHQKI IEEAPAVIAE PSVFEEMERA
TRIAVA_ACCase        LEVQLLADQY GNAISLFGRD CSIQRRHQKI IEEAPAVIAE PEVFEAMEKA
220O                 LEVQLLADQY GNAISLFGRD CSIQRRHQKI IEEAPAVIAE PDVFENMEKA
319                  LEVQLLADQY GNAISLFGRD CSIQRRHQKI IEEAPAVIAE PDVFENMEKA
419                  LEVQLLADQY GNAISLFGRD CSIQRRHQKI IEEAPAVIAE PDVFENMEKA
520                  LEVQLLADQY GNAISLFGRD CSIQRRHQKI IEEAPAVIAE PDVFENMEKA
619                  LEVQLLADQY GNAISLFGRD CSIQRRHQKI IEEAPAVIAE PDVFENMEKA

                     401                                              450
BEMITA_QJQ31013      AVRLAKMVGY VSAGTVEYLY DPSEGQYYFL ELNPRLQVEH PCTEMVADVN
MYZUPE_XP_022181497  AVRIAKMVGY VSAGTVEYLY DT.DGNYYFL ELNPRLQVEH PCTEMVSDVN
TRIAVA_ACCase        AVRLAKMVGY VSAGTVEYLY DPSEGQYYFL ELNPRLQVEH PCTEMVADVN
220O                 AVRLAKMVGY VSAGTVEYLY DPSEGQYFFL ELNPRLQVEH PCTEMVADVN
319                  AVRLAKMVGY VSAGTVEYLY DPSEGQYFFL ELNPRLQVEH PCTEMVADVN
419                  AVRLAKMVGY VSAGTVEYLY DPSEGQYFFL ELNPRLQVEH PCTEMVADVN
520                  AVRLAKMVGY VSAGTVEYLY DPSEGQYFFL ELNPRLQVEH PCTEMVADVN
619                  AVRLAKMVGY VSAGTVEYLY DPSEGQYFFL ELNPRLQVEH PCTEMVADVN

                     451                                              500
BEMITA_QJQ31013      LPAAQLQVAM GLQLHCIKDI RVLYGESPWG DSLIDFDQPR HKPQPWGHVI
MYZUPE_XP_022181497  LPAAQLQISM GLALNCIKDI RLLYSESAWG DSYIDFDAPR HKPHPWGHVI
TRIAVA_ACCase        LPAAQLQIAM GLPLNCIKDI RLLYGESPWG DNLIDFDEPR HKPQPWGHVI
220O                 LPAAQLQIAM GLQLNCIKDI RLLYGESPWG DNYIDFDEPR HKPQPWGHVI
319                  LPAAQLQIAM GLQLNCIKDI RLLYGESPWG DNYIDFDEPR HKPQPWGHVI
419                  LPAAQLQIAM GLQLNCIKDI RLLYGESPWG DNYIDFDEPR HKPQPWGHVI
520                  LPAAQLQIAM GLQLNCIKDI RLLYGESPWG DNYIDFDEPR HKPQPWGHVI
619                  LPAAQLQIAM GLQLNCIKDI RLLYGESPWG DNYIDFDEPR HKPQPWGHVI

                     501                                              550
BEMITA_QJQ31013      AARITSENPD EGFKPSSGTV QELNFRSSKN VWGYFSVAAS GGLHEFADSQ
MYZUPE_XP_022181497  AARITSENPD EGFKPSSGTV QELNFRSSKN VWGYFSVAAS GGLHEFADSQ
TRIAVA_ACCase        AARITSENPD EGFKPSSGTV QELNFRSSKN VWGYFSVAAS GGLHEFADSQ
220O                 AARITSENPD EGFKPSSGTV QELNFRSSKN VWGYFSVAAS GGLHEFADSQ
319                  AARITSENPD EGFKPSSGTV QELNFRSSKN VWGYFSVAAS GGLHEFADSQ
419                  AARITSENPD EGFKPSSGTV QELNFRSSKN VWGYFSVAAS GGLHEFADSQ
```

```
520                       AARITSENPD EGFKPSSGTV QELNFRSSKN VWGYFSVAAS GGLHEFADSQ
619                       AARITSENPD EGFKPSSGTV QELNFRSSKN VWGYFSVAAS GGLHEFADSQ

                          551                                                600
BEMITA_QJQ31013           FGHCFSWGEN REQARENLVI ALKELSIRGD FRTTVEYLIT LLETESFQIN
MYZUPE_XP_022181497       FGHCFSWGEN REQARENLVI ALKELSIRGD FRTTVEYLIT LLETESFQSN
TRIAVA_ACCase             FGHCFSWGEN REQARENLVI ALKELSIRGD FRTTVEYLIT LLETESFQIN
220O                      FGHCFSWGEN REQARENLVI ALKELSIRGD FRTTVEYLIT LLETESFQIN
319                       FGHCFSWGEN REQARENLVI ALKELSIRGD FRTTVEYLIT LLETESFQIN
419                       FGHCFSWGEN REQARENLVI ALKELSIRGD FRTTVEYLIT LLETESFQIN
520                       FGHCFSWGEN REQARENLVI ALKELSIRGD FRTTVEYLIT LLETESFQIN
619                       FGHCFSWGEN REQARENLVI ALKELSIRGD FRTTVEYLIT LLETESFQIN

                          601                                                650
BEMITA_QJQ31013           TIDTAWLDVL IAEKVQSEKP DILLGVMCGA LHIADRRVTD AFQNFQTSLE
MYZUPE_XP_022181497       TIDTAWLDLL ISERVQSEKP DIFLGVICGG LHIADRKISE SFQNFQTSLE
TRIAVA_ACCase             TIDTAWLDVL IAQKVPSEKP DILLGVMCGA LHIADRKVTD AFQNFQTSLE
220O                      TIDTAWLDLL ISERVQSEKP DVLLGVMCGA LHIADRKVCD AFQNFQTSLE
319                       TIDTAWLDLL ISERVQSEKP DVLLGVMCGA LHIADRKVCD AFQNFQTSLE
419                       TIDTAWLDLL ISERVQSEKP DVLLGVMCGA LHIADRKVCD AFQNFQTSLE
520                       TIDTAWLDLL ISERVQSEKP DVLLGVMCGA LHIADRKVCD AFQNFQTSLE
619                       TIDTAWLDLL ISERVQSEKP DVLLGVMCGA LHIADRKVCD AFQNFQTSLE

                          651                                                700
BEMITA_QJQ31013           RGQIQGSNTL DHNVQVELIN DGLKYKVHAT KSGPNSYFLV MNGSFKEIEL
MYZUPE_XP_022181497       RGQVLSANTL DHHVSVELIN GGYKYKVQVT KSGLNSYFLI MNGSFKEIEV
TRIAVA_ACCase             RGQIQGSNTL DHHLSVELIH DGLKYRVHAT KSGANSYFLV MNGSFKEIEL
220O                      RGQIQGCNTL DHNVQVELIH DGLKYKVHAT KSGLNSYFLV MNGSFKEIEL
319                       RGQIQGCNTL DHNVQVELIH DGLKYKVHAT KSGLNSYFLV MNGSFKEIEL
419                       RGQIQGCNTL DHNVQVELIH DGLKYKVHAT KSGLNSYFLV MNGSFKEIEL
520                       RGQIQGCNTL DHNVQVELIH DGLKYKVHAT KSGLNSYFLV MNGSFKEIEL
619                       RGQIQGCNTL DHNVQVELIH DGLKYKVHAT KSGLNSYFLV MNGSFKEIEL

                          701                                                750
BEMITA_QJQ31013           HRLSDGGILL SVDSSSFTTY MREEVDRYRI VIGNQTCVFE KENDPSLLRS
MYZUPE_XP_022181497       HRLSDGGILL SLDGSSFTTY MREEVDRYRI VIGNQTCVFD KENDPSLFRS
TRIAVA_ACCase             HRLSDG.... .......... ...EVDRYRI VIGNQTCVFE KENDPSLLRS
220O                      HRLSDGGILL SVDGSSFTTY MREEVDRYRI VIGNLTCVFE KENDPSLLRS
319                       HRLSDGGILL SVDGSSFTTY MREEVDRYRI VIGNLTCVFE KENDPSLLRS
419                       HRLSDGGILL SVDGSSFTTY MREEVDRYRI VIGNLTCVFE KENDPSLLRS
520                       HRLSDGGILL SVDGSSFTTY MREEVDRYRI VIGNLTCVFE KENDPSLLRS
619                       HRLSDGGILL SVDGSSFTTY MREEVDRYRI VIGNLTCVFE KENDPSLLRS

                          751                                                800
BEMITA_QJQ31013           PSAGKLLSFL IEDGGHVSKG QAYAEIEVMK MVMTLTASEN GNVTFAKRPG
MYZUPE_XP_022181497       PSAGKLISFL IEDGGQVKKG QPYAEIEVMK MVMTLTATEN GRVYYSKRPG
TRIAVA_ACCase             PSAGKLISFL IEDGGHVAKG AAYAEIEVMK MVMTLTANES GLVTFAKRPG
220O                      PSAGKLISYL IEDGGHVSKG QAYAEIEVMK MVMTLTANES GLVTFAKRPG
319                       PSAGKLISYL IEDGGHVSKG QAYAEIEVMK MVMTLTANES GLVTFAKRPG
419                       PSAGKLISYL IEDGGHVSKG QAYAEIEVMK MVMTLTANES GLVTFAKRPG
520                       PSAGKLISYL IEDGGHVSKG QAYAEIEVMK MVMTLTANES GLVTFAKRPG
619                       PSAGKLISYL IEDGGHVSKG QAYAEIEVMK MVMTLTANES GLVTFAKRPG

                          801                                                850
BEMITA_QJQ31013           AVLDAGSLIA TLELDDPSLV TKALDYKGQF PELDVSTPTV GEKLNHAHNH
MYZUPE_XP_022181497       AVLDAGSLIA TLELDDPSLV TKAIEYKGQF LELDGTSHIY GESLNNIHTC
TRIAVA_ACCase             AVLDAGSLIA TLELDDISLV TKALDYKGQF PELDVSTPMV GDKLNHAHNH
220O                      AVLDAGSLIG TLELDDPSLV SKALDYKGEF PELDVSTPMV GEKLNHAHNH
319                       AVLDAGSLIG TLELDDPSLV SKALDYKGEF PELDVSTPMV GEKLNHAHNH
```

```
419                    AVLDAGSLIG TLELDDPSLV SKALDYKGEF PELDVSTPMV GEKLNHAHNH
520                    AVLDAGSLIG TLELDDPSLV SKALDYKGEF PELDVSTPMV GEKLNHAHNH
619                    AVLDAGSLIG TLELDDPSLV SKALDYKGEF PELDVSTPMV GEKLNHAHNH

                       851                                                900
BEMITA_QJQ31013        YRQMLDNILA GYCLPDPYHL VRLREVIEKF MSSLRDPSLP LLELQEVISS
MYZUPE_XP_022181497    YRGMLDNILA GYCLPEPYHL VRLREVIEKF MNSLRDPSLP LLELQEVISS
TRIAVA_ACCase          YRQMLDNILA GYCLPDPYHL VRLREVIEKF MSSLRDPSLP LLELQEVISS
220O                   YRQMLDNILA GFCLPDPYHL VRLREVIEKF MSSLRDPSLP LLELQEVISS
319                    YRQMLDNILA GFCLPDPYHL VRLREVIEKF MSSLRDPSLP LLELQEVISS
419                    YRQMLDNILA GFCLPDPYHL VRLREVIEKF MSSLRDPSLP LLELQEVISS
520                    YRQMLDNILA GFCLPDPYHL VRLREVIEKF MSSLRDPSLP LLELQEVISS
619                    YRQMLDNILA GFCLPDPYHL VRLREVIEKF MSSLRDPSLP LLELQEVISS

                       901                                                950
BEMITA_QJQ31013        ISGRIPISVE KKIRKLMTLY ERNITSVLAQ FPSQQIASVI DSHAATLQKR
MYZUPE_XP_022181497    ISGRIPKAVD KKIKSLMKLY ERNITSVLAQ FPSQQIAAII DGHAATLQKR
TRIAVA_ACCase          ISGRIPISVE KKIRKLMTLY ERNITSVLAQ FPSQQIASVI DSHAATLQKR
220O                   ISGRIPISVE KKIRKLMTLY ERNITSVLAQ FPSQQIAGVI DSHAATLQKR
319                    ISGRIPISVE KKIRKLMTLY ERNITSVLAQ FPSQQIAGVI DSHAATLQKR
419                    ISGRIPISVE KKIRKLMTLY ERNITSVLAQ FPSQQIAGVI DSHAATLQKR
520                    ISGRIPISVE KKIRKLMTLY ERNITSVLAQ FPSQQIAGVI DSHAATLQKR
619                    ISGRIPISVE KKIRKLMTLY ERNITSVLAQ FPSQQIAGVI DSHAATLQKR

                       951                                               1000
BEMITA_QJQ31013        SDRDGFFLTT QGIVQLVQRY RNGIRGRMKS AVHELLRQYY EVESQFQQGH
MYZUPE_XP_022181497    TDRDSFFQTT QGIVQLVQRY RNGIRGRMKS AVHELLKQYY EVESQFQHGS
TRIAVA_ACCase          SDRDGFFLTT QGIVQLVQRY RNGIRGRMKS AVHELLRQYY EVESQFQQGH
220O                   SDRDGFFLTT QGIVQLVQRY RNGIRGRMKS AVHELLRQYY EVESQFQQGH
319                    SDRDGFFLTT QGIVQLVQRY RNGIRGRMKS AVHELLRQYY EVESQFQQGH
419                    SDRDGFFLTT QGIVQLVQRY RNGIRGRMKS AVHELLRQYY EVESQFQQGH
520                    SDRDGFFLTT QGIVQLVQRY RNGIRGRMKS AVHELLRQYY EVESQFQQGH
619                    SDRDGFFLTT QGIVQLVQRY RNGIRGRMKS AVHELLRQYY EVESQFQQGH

                       1001                                              1050
BEMITA_QJQ31013        YDKCVTAIRE KFKDDMAAVT GTIFSHGQVA KKNMLVTMLI DHLWSNEPGL
MYZUPE_XP_022181497    YDKCATALRD RYKDDMAAVT STIFSHTQVA KKNMLVTMLI DHLWSNEPGL
TRIAVA_ACCase          YDKCVTAIRE KFKDDMAAVV GTIFSHGQVA KKNMLVTMLI DHLWSNEPGL
220O                   YDKCVMAIRE KFKDDMAAVT STIFSHGQVA KKNMLVTMLI DHLWSNEPGL
319                    YDKCVMAIRE KFKDDMAAVT STIFSHGQVA KKNMLVTMLI DHLWSNEPGL
419                    YDKCVMAIRE KFKDDMAAVT STIFSHGQVA KKNMLVTMLI DHLWSNEPGL
520                    YDKCVMAIRE KFKDDMAAVT STIFSHGQVA KKNMLVTMLI DHLWSNEPGL
619                    YDKCVMAIRE KFKDDMAAVT STIFSHGQVA KKNMLVTMLI DHLWSNEPGL

                       1051                                              1100
BEMITA_QJQ31013        TDELATTLNE LTSLNRSEHS RVALRARQVL IAAHQPAYEL RHNQMESIFL
MYZUPE_XP_022181497    TDELAATLNE LTSLNRSEHS RVALRARQVL IAAHQPAYEL RHNQMESIFL
TRIAVA_ACCase          TDELATTLNE LTSLNRSEHS RVALRARQVL IAAHQPAYEL RHNQMESIFL
220O                   TDELATTLNE LTSLNRSEHS RVALRSRQVL IAAHQPAYEL RHNQMESIFL
319                    TDELATTLNE LTSLNRSEHS RVALRSRQVL IAAHQPAYEL RHNQMESIFL
419                    TDELATTLNE LTSLNRSEHS RVALRSRQVL IAAHQPAYEL RHNQMESIFL
520                    TDELATTLNE LTSLNRSEHS RVALRSRQVL IAAHQPAYEL RHNQMESIFL
619                    TDELATTLNE LTSLNRSEHS RVALRSRQVL IAAHQPAYEL RHNQMESIFL

                       1101                                              1150
BEMITA_QJQ31013        SAVDMYGHDF HPENLQKLIQ SETSIFDILH DFFYHTNRAV CNAALEVYVR
MYZUPE_XP_022181497    SAVDMYGHDF HPENLQKLIQ SETSIFDILH DFFYHSNRAV CNAALEVYVR
TRIAVA_ACCase          SAVDMYGHDF HPENLQKLIQ SETSIFDILH DFFYHTNRAV CNAALEVYVR
220O                   SAVDMYGHDF HPENLQKLIQ SETSIFDILH DFFYHTNRAV CNAALEVYVR
```

```
319                   SAVDMYGHDF HPENLQKLIQ SETSIFDILH DFFYHTNRAV CNAALEVYVR
419                   SAVDMYGHDF HPENLQKLIQ SETSIFDILH DFFYHTNRAV CNAALEVYVR
520                   SAVDMYGHDF HPENLQKLIQ SETSIFDILH DFFYHTNRAV CNAALEVYVR
619                   SAVDMYGHDF HPENLQKLIQ SETSIFDILH DFFYHTNRAV CNAALEVYVR

                      1151                                                1200
BEMITA_QJQ31013       RVYISYELTC LKHLELTEEV PLVQFQFLLP SSHPNRQRVT DSASPGRDAP
MYZUPE_XP_022181497   RVYISYDLTC LQHLELSGEI PLVHFQFLLP SSHPNRQQNK INSGAN...G
TRIAVA_ACCase         RVYISYDLTC LQHLELSGEI PLVQFQFLLP SSHPNRQKVT DSSPIKADTP
220O                  RVYISYELTC LQHLELSGEV PLVQFQFLLP SSHPNRQKVT DPSSPIIAD.
319                   RVYISYELTC LQHLELSGEV PLVQFQFLLP SSHPNRQKVT DPSSPIIAD.
419                   RVYISYELTC LQHLELSGEV PLVQFQFLLP SSHPNRQKVT DPSSPIIAD.
520                   RVYISYELTC LQHLELSGEV PLVQFQFLLP SSHPNRQKVT DPSSPIIAD.
619                   RVYISYELTC LQHLELSGEV PLVQFQFLLP SSHPNRQKVT DPSSPIIAD.

                      1201                                                1250
BEMITA_QJQ31013       ..ETDMTASA APTVIHSYQR TGCMAAFESF DQFESYYDEI LDLLDEL.SP
MYZUPE_XP_022181497   SENLESPTKT PLPYIPTYQR TGCMAAFESF TQFEQYFDEI LDIMEDLSSP
TRIAVA_ACCase         PPEIEAGQSS APTIIHSYQR TGCMAAFESF DQFESYYDEI LDLLDDLMSP
220O                  SPETEEPPSS TPTIIHSYQR TGCMAAFESF DQFESYYDEI LDLLDEL.SP
319                   SPETEEPPSS TPTIIHSYQR TGCMAAFESF DQFESYYDEI LDLLDEL.SP
419                   SPETEEPPSS TPTIIHSYQR TGCMAAFESF DQFESYYDEI LDLLDEL.SP
520                   SPETEEPPSS TPTIIHSYQR TGCMAAFESF DQFESYYDEI LDLLDEL.SP
619                   SPETEEPPSS TPTIIHSYQR TGCMAAFESF DQFESYYDEI LDLLDEL.SP

                      1251                                                1300
BEMITA_QJQ31013       STVSPRIMEA LESGSESRMS TSINVSLSVD .TQRPAGGEE GLQVEPIHIL
MYZUPE_XP_022181497   VYVSPRIIDA LESGSESRLS SSLNVSLSLG DQRPPD..QE NVEIEPCHIL
TRIAVA_ACCase         STVSPRIMEA LESGSESRMS TSINVSLSVS DNPRPQGLEE GLQVEPIHIL
220O                  STISPRIMEA LESGSESRMS TSINVSLSVG DNPRPPGLED GLQVEPIHIL
319                   STISPRIMEA LESGSESRMS TSINVSLSVG DNPRPPGLED GLQVEPIHIL
419                   STISPRIMEA LESGSESRMS TSINVSLSVG DNPRPPGLED GLQVEPIHIL
520                   STISPRIMEA LESGSESRMS TSINVSLSVG DNPRPPGLED GLQVEPIHIL
619                   STISPRIMEA LESGSESRMS TSINVSLSVG DNPRPPGLED GLQVEPIHIL

                      1301                                                1350
BEMITA_QJQ31013       CIAVKDNGDL EDDKLSKMFG DFCAKNRDEL KEKSIRRITF LALNRRQFPK
MYZUPE_XP_022181497   CIAMKDTGNM EDDKLGKMYE EFCQQRREEL KKRSIRRITF LALNKRQFPK
TRIAVA_ACCase         CIAVKDNGDL EDDKLSKLLG DFCARHRDEL KEKSIRRITF LALNRRQFPK
220O                  CIAVKDNGDL EDEKLSKLLG DFCAKHREEL KEKSIRRITF LALNRRQFPK
319                   CIAVKDNGDL EDEKLSKLLG DFCAKHREEL KEKSIRRITF LALNRRQFPK
419                   CIAVKDNGDL EDEKLSKLLG DFCAKHREEL KEKSIRRITF LALNRRQFPK
520                   CIAVKDNGDL EDEKLSKLLG DFCAKHREEL KEKSIRRITF LALNRRQFPK
619                   CIAVKDNGDL EDEKLSKLLG DFCAKHREEL KEKSIRRITF LALNRRQFPK

                      1351                                                1400
BEMITA_QJQ31013       LFTFRNCDNF VEDRIYRHLE PGMAFQLELN RMKTYHLEAL PTSNRKMYLY
MYZUPE_XP_022181497   LFTYRNYEDF AEDRIYRHLE PGMAFQLELN RMRTYELEAL PTSNRKMYLY
TRIAVA_ACCase         LFTFRNCDNF VEDRIYRHLE PGMAFQLELN RMKTYHLEAL PTSNRKMYLY
220O                  LFTFRNCDNF VEDRIYRHLE PGMAFQLELN RMKTYHLEAL PTSNRKMYLY
319                   LFTFRNCDNF VEDRIYRHLE PGMAFQLELN RMKTYHLEAL PTSNRKMYLY
419                   LFTFRNCDNF VEDRIYRHLE PGMAFQLELN RMKTYHLEAL PTSNRKMYLY
520                   LFTFRNCDNF VEDRIYRHLE PGMAFQLELN RMKTYHLEAL PTSNRKMYLY
619                   LFTFRNCDNF VEDRIYRHLE PGMAFQLELN RMKTYHLEAL PTSNRKMYLY

                      1401                                                1450
BEMITA_QJQ31013       LGKAKVAKGQ EVTDYRFFIR SIIRHSDLIT KEASFEYLQN EGERVLLEAM
MYZUPE_XP_022181497   LGKAKVPRGQ VVTDYRFFIR SIIRHQDLIT KEASFEYLQN EGERVLLEAM
TRIAVA_ACCase         LGKAKVAKGQ EVTDYRFFIR SIIRHSDLIT KEASFEYLQN EGERVLLEAM
```

```
2200                    LGRAKVAKGQ EVTDFRFFIR SIIRHSDLIT KEASFEYLQN EGERVLLEAM
319                     LGRAKVAKGQ EVTDFRFFIR SIIRHSDLIT KEASFEYLQN EGERVLLEAM
419                     LGRAKVAKGQ EVTDFRFFIR SIIRHSDLIT KEASFEYLQN EGERVLLEAM
520                     LGRAKVAKGQ EVTDFRFFIR SIIRHSDLIT KEASFEYLQN EGERVLLEAM
619                     LGRAKVAKGQ EVTDFRFFIR SIIRHSDLIT KEASFEYLQN EGERVLLEAM

                        1451                                              1500
BEMITA_QJQ31013         DELEVAFSHP LAKRTDCNHI FLNFVPTVIM DSAKIEESVT NMVMRYGPRL
MYZUPE_XP_022181497     DELEVAFSHP HARRTDCNHI FLNFVPTVIM DPAKIKESVT NMVMRYGPRL
TRIAVA_ACCase           DELEVAFSHP LAKRTDCNHI FLNFVPTVIM DSAKIEESVT NMVMRYGPRL
2200                    DELEVAFSHP LARRTDCNHI FLNFVPTVIM DSAKIEESVT NMVMRYGARL
319                     DELEVAFSHP LARRTDCNHI FLNFVPTVIM DSAKIEESVT NMVMRYGARL
419                     DELEVAFSHP LARRTDCNHI FLNFVPTVIM DSAKIEESVT NMVMRYGARL
520                     DELEVAFSHP LARRTDCNHI FLNFVPTVIM DSAKIEESVT NMVMRYGARL
619                     DELEVAFSHP LARRTDCNHI FLNFVPTVIM DSAKIEESVT NMVMRYGARL

                        1501                                              1550
BEMITA_QJQ31013         WKLRVLQAEL RMTIRASPNA KTTNVRLCLA NDSGYYLDIC LYKEVVDPKT
MYZUPE_XP_022181497     WKLRVLQAEL RMTIRPSPTS KTSNVRLSLA NGSGYHLDIC LYKEITDSKL
TRIAVA_ACCase           WKLRVLQAEL RMTIRASPNS KTTNVRLCLA NDSGYYLDIC LYKEVVDPKT
2200                    WKLRVLQAEL RMTIRTSPNS KTTNVRLCLA NDSGYYLDIC LYKEVVDPKT
319                     WKLRVLQAEL RMTIRTSPNS KTTNVRLCLA NDSGYYLDIC LYKEVVDPKT
419                     WKLRVLQAEL RMTIRTSPNS KTTNVRLCLA NDSGYYLDIC LYKEVVDPKT
520                     WKLRVLQAEL RMTIRTSPNS KTTNVRLCLA NDSGYYLDIC LYKEVVDPKT
619                     WKLRVLQAEL RMTIRTSPNS KTTNVRLCLA NDSGYYLDIC LYKEVVDPKT

                        1551                                              1600
BEMITA_QJQ31013         GIIKLESYGS KQGPLHGLPV ATPYVTKDYL QQKRFQAQSA GTTYCHDIPD
MYZUPE_XP_022181497     GMIKFESYES KQGPLHGLPV STPYVTKDFL QQKRFQAQSA GTTYVYDIPD
TRIAVA_ACCase           GIIKLESYGS KQGPVHGLPV SLPYVTKDYL QQKRFQAQSA GTTYCHDIPD
2200                    GIIKLESYGS KQGPLHGLPT AIPYVTKDYL QQKRFQAQSA GTTYCHDIPD
319                     GIIKLESYGS KQGPLHGLPT AIPYVTKDYL QQKRFQAQSA GTTYCHDIPD
419                     GIIKLESYGS KQGPLHGLPT AIPYVTKDYL QQKRFQAQSA GTTYCHDIPD
520                     GIIKLESYGS KQGPLHGLPT AIPYVTKDYL QQKRFQAQSA GTTYCHDIPD
619                     GIIKLESYGS KQGPLHGLPT AIPYVTKDYL QQKRFQAQSA GTTYCHDIPD

                        1601                                              1650
BEMITA_QJQ31013         MFRQMVERQW KEHIDQRPDD GIVKPAQLMD FAELVLEEDH LVEQKRLPGE
MYZUPE_XP_022181497     MFRQMIETLW QEYILEHPND GVLKSSLVFD YVELVVEDNH LIEQKRFSGE
TRIAVA_ACCase           MFRQMVERQW KEHIEQRPND GIVKPSQLMD FVELVMEEDH LIEVKRLSGE
2200                    MFRQMVERQW KEFIEQRPND GIVKPSQLMD YVELVLEDDH LVEQKRLAGE
319                     MFRQMVERQW KEFIEQRPND GIVKPSQLMD YVELVLEDDH LVEQKRLAGE
419                     MFRQMVERQW KEFIEQRPND GIVKPSQLMD YVELVLEDDH LVEQKRLAGE
520                     MFRQMVERQW KEFIEQRPND GIVKPSQLMD YVELVLEDDH LVEQKRLAGE
619                     MFRQMVERQW KEFIEQRPND GIVKPSQLMD YVELVLEDDH LVEQKRLAGE

                        1651                                              1700
BEMITA_QJQ31013         NNVGMVAWRI TLNTPEYPDG RDIIVIANDI TVRIGSFGPE EDLVFDLASK
MYZUPE_XP_022181497     NTAGMVAWRF TMHTPEYPSG RDIIVIANDL TVNIGSFGPQ EDIVFDLASK
TRIAVA_ACCase           NNVGMVAWRI TLNTPEYPDG RDIIVIANDI TVRIGSFGPE EDIVFDLASK
2200                    NTIGMVAWRI TLNTPEYPDG REIIVIANDI TVRIGSFGPE EDLVFDLASK
319                     NTIGMVAWRI TLNTPEYPDG REIIVIANDI TVRIGSFGPE EDLVFDLASK
419                     NTIGMVAWRI TLNTPEYPDG REIIVIANDI TVRIGSFGPE EDLVFDLASK
520                     NTIGMVAWRI TLNTPEYPDG REIIVIANDI TVRIGSFGPE EDLVFDLASK
619                     NTIGMVAWRI TLNTPEYPDG REIIVIANDI TVRIGSFGPE EDLVFDLASK

                        1701                                              1750
BEMITA_QJQ31013         IARQRKIPRI YIAANSGARI GLAEEVKSLF RVAWEDPDEP DKGFKYLYLS
MYZUPE_XP_022181497     EARRKKIPRI YISANSGARI GLAEEIKSLF NVAWEDPSDP EKGFKYLYLT
```

```
TRIAVA_ACCase           IARARKIPRI YIAANSGARI GLAEEVKSLF RVAWEDPDEP DKGFKYLYLS
2200                    IARQMKVPRI YIAANSGARI GLAEEVKSLF RVAWEDPDEP DKGLKYLYLS
319                     IARQMKVPRI YIAANSGARI GLAEEVKSLF RVAWEDPDEP DKGLKYLYLS
419                     IARQMKVPRI YIAANSGARI GLAEEVKSLF RVAWEDPDEP DKGLKYLYLS
520                     IARQMKVPRI YIAANSGARI GLAEEVKSLF RVAWEDPDEP DKGLKYLYLS
619                     IARQMKVPRI YIAANSGARI GLAEEVKSLF RVAWEDPDEP DKGLKYLYLS

                        1751                                              1800
BEMITA_QJQ31013         TEDFTKISSC NSVHAILIED EGEARYKITD IIGKEDGLGV ENLRYAGMIA
MYZUPE_XP_022181497     PDDYGKLAGQ NSVEAELIED EGEPRYKLTD IIGKDFGYGV ENLKFAGMIA
TRIAVA_ACCase           TEDFTKISSL NSVHAILIED EGEARYKITD IIGKEDGLGV ENLRYAGMIA
2200                    TEDFIKISAC NSVRAILIED EGEARYKITD IIGKEDGLGV ENLRYAGMIA
319                     TEDFIKISAC NSVRAILIED EGEARYKITD IIGKEDGLGV ENLRYAGMIA
419                     TEDFIKISAC NSVRAILIED EGEARYKITD IIGKEDGLGV ENLRYAGMIA
520                     TEDFIKISAC NSVRAILIED EGEARYKITD IIGKEDGLGV ENLRYAGMIA
619                     TEDFIKISAC NSVRAILIED EGEARYKITD IIGKEDGLGV ENLRYAGMIA

                        1801                                              1850
BEMITA_QJQ31013         GETSEAYREI VTISMVSCRA IGIGAYLVRL GQRVIQVENS HIILTGYSAL
MYZUPE_XP_022181497     GETSRAYQDI VTISMVTCRA IGIGAYLVRL GQRVIQIENS HIILTGYSAL
TRIAVA_ACCase           GETSEAYREI VTISMVSCRA IGIGAYLVRL GQRVIQVENS HIILTGYSAL
2200                    GETSEAYREI VTISMVSCRA IGIGAYLVRL GQRVIQVENS HIILTGYSAL
319                     GETSEAYREI VTISMVSCRA IGIGAYLVRL GQRVIQVENS HIILTGYSAL
419                     GETSEAYREI VTISMVSCRA IGIGAYLVRL GQRVIQVENS HIILTGYSAL
520                     GETSEAYREI VTISMVSCRA IGIGAYLVRL GQRVIQVENS HIILTGYSAL
619                     GETSEAYREI VTISMVSCRA IGIGAYLVRL GQRVIQVENS HIILTGYSAL

                        1851                                              1900
BEMITA_QJQ31013         NKLLGREVYA SNNQLGGIQI MYNNGVSHKT EPRDLDGIYS IVKWLSYIPK
MYZUPE_XP_022181497     NKLLGREVYA SNNQLGGIQI MYNNGVSHKT EARDLDGVYR ILKWLSYIPK
TRIAVA_ACCase           NKLLGREVYA SNNQLGGIQI MHNNGVSHKT EPRDLDGIYS IVKWLAFVPK
2200                    NKLLGREVYA SNNQLGGIQI MYNNGISHKT EPRDLDGIYS IVKWLGYIPK
319                     NKLLGREVYA SNNQLGGIQI MYNNGISHKT EPRDLDGIYS IVKWLGYIPK
419                     NKLLGREVYA SNNQLGGIQI MYNNGISHKT EPRDLDGIYS IVKWLGYIPK
520                     NKLLGREVYA SNNQLGGIQI MYNNGISHKT EPRDLDGIYS IVKWLGYIPK
619                     NKLLGREVYA SNNQLGGIQI MYNNGISHKT EPRDLDGIYS IVKWLGYIPK

                        1901                                              1950
BEMITA_QJQ31013         DKLSPVPVIK PADPIDREVG YMPTKTPYDP RWMLAGRYSP .NNSNEWESG
MYZUPE_XP_022181497     TKESPLPVIK SVDSVERDID YVPTKVPYDP RWMIAGKE.. .DTNGHWESG
TRIAVA_ACCase           DKMSPVPVLK PADPVDREVG YMPTKTPYDP RWMLAGHFSF LDSSNEWESG
2200                    DKYSPVPVIK PADPVDREVG YMPTKTPYDP RWMIAGRYSP .NNSNEWESG
319                     DKYSPVPVIK PADPVDREVG YMPTKTPYDP RWMIAGRYSP .NNSNEWESG
419                     DKYSPVPVIK PADPVDREVG YMPTKTPYDP RWMIAGRYSP .NNSNEWESG
520                     DKYSPVPVIK PADPVDREVG YMPTKTPYDP RWMIAGRYSP .NNSNEWESG
619                     DKYSPVPVIK PADPVDREVG YMPTKTPYDP RWMIAGRYSP .NNSNEWESG

                        1951                                              2000
BEMITA_QJQ31013         FFDEGSWEEV MQPWAQTVVV GRARLGGIPM GVIAVETRTV EVKLPADPAN
MYZUPE_XP_022181497     FFDKGSWDEI MQPWAQTVVC GRARLGGIPV GVIAVETRTV EVTLPADPAN
TRIAVA_ACCase           FFDEGTWEEV MQPWAQTVVV GRARLGGIPM GVIAVETRTV ELKWPADPAN
2200                    FFDEGSWEEV MQPWAQTVVV GRARLGGIPM GVIAVETRTV EVKLPADPAN
319                     FFDEGSWEEV MQPWAQTVVV GRARLGGIPM GVIAVETRTV EVKLPADPAN
419                     FFDEGSWEEV MQPWAQTVVV GRARLGGIPM GVIAVETRTV EVKLPADPAN
520                     FFDEGSWEEV MQPWAQTVVV GRARLGGIPM GVIAVETRTV EVKLPADPAN
619                     FFDEGSWEEV MQPWAQTVVV GRARLGGIPM GVIAVETRTV EVKLPADPAN

                        2001                                              2050
BEMITA_QJQ31013         LDSEAKTLSQ AGQVWFPDSA YKTAQAIKDF EHEDLPLIIF ANWRGFSGGM
```

```
MYZUPE_XP_022181497    LDSESKTVSQ AGQVWFPDSA YKTSQAIKDF AHEDLPLFIF ANWRGFSGGM
TRIAVA_ACCase          LDSEAKTLSQ AGQVWFPDSA YKTAQAIKDF QHEDLPLIIF ANWRGFSGGM
2200                   LDSEAKTLSQ AGQVWFPDSA FKTAQAIKDF EHEDLPLIIF ANWRGFSGGM
319                    LDSEAKTLSQ AGQVWFPDSA FKTAQAIKDF EHEDLPLIIF ANWRGFSGGM
419                    LDSEAKTLSQ AGQVWFPDSA FKTAQAIKDF EHEDLPLIIF ANWRGFSGGM
520                    LDSEAKTLSQ AGQVWFPDSA FKTAQAIKDF EHEDLPLIIF ANWRGFSGGM
619                    LDSEAKTLSQ AGQVWFPDSA FKTAQAIKDF EHEDLPLIIF ANWRGFSGGM

                       2051                                      A2083V    2100
BEMITA_QJQ31013        KDMYEQVMKF GAYIVDELRV YKQPVIIYIP PNGELRGGAW AVVDPTINPR
MYZUPE_XP_022181497    KDMYEQIMKF GAYIVDELRQ YNQPIITYIP PFGELRGGAW AVVDTTINPR
TRIAVA_ACCase          KDMYEQVMKF GAYIVDELRL YKQPVIIYIP PNGELRGGAW AVVDPTINPR
2200                   KDMYEQVMKF GAYIVDELRV YKQPVIIYIP PNGELRGGAW VVVDPTINPR
319                    KDMYEQVMKF GAYIVDELRV YKQPVIIYIP PNGELRGGAW AVVDPTINPR
419                    KDMYEQVMKF GAYIVDELRV YKQPVIIYIP PNGELRGGAW AVVDPTINPR
520                    KDMYEQVMKF GAYIVDELRV YKQPVIIYIP PNGELRGGAW AVVDPTINPR
619                    KDMYEQVMKF GAYIVDELRV YKQPVIIYIP PNGELRGGAW AVVDPTINPR

                       2101                                             2150
BEMITA_QJQ31013        HMEMYADPES RGGVLEPEGI VEIKFREKDI LKTMHRIDQV IVPLKQRLSS
MYZUPE_XP_022181497    HIEMYADPDS RGGVLEPEGI VEIKFREKDI LKSINRIDTN ILSLKAN...
TRIAVA_ACCase          YMEMYADPES RGGVLEPEGI VEIKFREKDI LKTMHRIDQI IVPLKQKLAN
2200                   HMEMYADPES RGGVLEPEGI VEIKFREKDI LKTMHRIDQV IVPLKQKLAV
319                    HMEMYADPES RGGVLEPEGI VEIKFREKDI LKTMHRIDQV IVPLKQKLAV
419                    HMEMYADPES RGGVLEPEGI VEIKFREKDI LKTMHRIDQV IVPLKQKLAV
520                    HMEMYADPES RGGVLEPEGI VEIKFREKDI LKTMHRIDQV IVPLKQKLAV
619                    HMEMYADPES RGGVLEPEGI VEIKFREKDI LKTMHRIDQV IVPLKQKLAV

                       2151                                             2200
BEMITA_QJQ31013        TDISPEEKAD VESRIVEREQ YLKPMYHQVA VHFADLHDTP ERMVEKGVIH
MYZUPE_XP_022181497    ASPTPEEAVE IEKNVAERIS VLKPIYHQVA IHFADLHDTP KCMLSKGVIK
TRIAVA_ACCase          PDISPEEKAE VENQIVEREQ YLKPMYHQVA VHFADLHDTP ERMMEKGVIH
2200                   PDLSPEEKAE VENQIVEREQ YLKPMYHQVA IHFADLHDTP ERMMEKGVIH
319                    PDLSPEEKAE VENQIVEREQ YLKPMYHQVA IHFADLHDTP ERMMEKGVIH
419                    PDLSPEEKAE VENQIVEREQ YLKPMYHQVA IHFADLHDTP ERMMEKGVIH
520                    PDLSPEEKAE VENQIVEREQ YLKPMYHQVA IHFADLHDTP ERMMEKGVIH
619                    PDLSPEEKAE VENQIVEREQ YLKPMYHQVA IHFADLHDTP ERMMEKGVIH

                       2201                                             2250
BEMITA_QJQ31013        DIVPWRKSRT ILHWRVKRLL LENQIKSNLL KVQPQMDDGQ AQAMLRRWFV
MYZUPE_XP_022181497    DIVQWKKSRN TLYWRLKRRL LQNQIQKVIT KSNDTIQDDV AYEMLRRWFV
TRIAVA_ACCase          DIVPWRKSRS IIHWRMKRLL LENQIKSNLI RVQPQLDDGQ AQAMLRRWFV
2200                   NIVPWRKSRT LLHWRVKRLL LENQIKCNLL KVQPQMDNGQ AQAMLRRWFV
319                    NIVPWRKSRT LLHWRVKRLL LENQIKCNLL KVQPQMDNGQ AQAMLRRWFV
419                    NIVPWRKSRT LLHWRVKRLL LENQIKCNLL KVQPQMDNGQ AQAMLRRWFV
520                    NIVPWRKSRT LLHWRVKRLL LENQIKCNLL KVQPQMDNGQ AQAMLRRWFV
619                    NIVPWRKSRT LLHWRVKRLL LENQIKCNLL KVQPQMDNGQ AQAMLRRWFV

                       2251                                             2300
BEMITA_QJQ31013        EDIGTTTAYL WENNESVVSW LMGQLS.PDG SISPNSIVAN NIRCVQRDAL
MYZUPE_XP_022181497    EDKGTTASYL WDNNQAVVQW LTSQLDESDG TIVADSLIGN NIKSVRKDAV
TRIAVA_ACCase          EDKGTTTAYL WENNESVVSW LMTQLS.PDG SISPHSIVAN NIRCVQRDAL
2200                   EDKGTTTAYL WENNESVVAW LMSQLS.PDG SISPNSIVAN NIRCVQRDAL
319                    EDKGTTTAYL WENNESVVAW LMSQLS.PDG SISPNSIVAN NIRCVQRDAL
419                    EDKGTTTAYL WENNESVVAW LMSQLS.PDG SISPNSIVAN NIRCVQRDAL
520                    EDKGTTTAYL WENNESVVAW LMSQLS.PDG SISPNSIVAN NIRCVQRDAL
619                    EDKGTTTAYL WENNESVVAW LMSQLS.PDG SISPNSIVAN NIRCVQRDAL

                       2301                                             2350
```

```
BEMITA_QJQ31013        INQIKTSMEE SPDVAQDAVV EMFQTLSASE RSEVLRKLSH LETISKPEPQ
MYZUPE_XP_022181497    INQVKSTIND TPEVTSDVIM GMFQSLSEMQ RLDLIHNLTQ ATSIGNVKLN
TRIAVA_ACCase          INQIKTSMEE SPDVAQDAVV EMFQALSSSE RSEVLRKLSH LEMINSSEPQ
220O                   INQIKTSMEE SPDVAQDAVV EMFQTLSANE RSEVLRKLSH LETISKPEPQ
319                    INQIKTSMEE SPDVAQDAVV EMFQTLSANE RSEVLRKLSH LETISKPEPQ
419                    INQIKTSMEE SPDVAQDAVV EMFQTLSANE RSEVLRKLSH LETISKPEPQ
520                    INQIKTSMEE SPDVAQDAVV EMFQTLSANE RSEVLRKLSH LETISKPEPQ
619                    INQIKTSMEE SPDVAQDAVV EMFQTLSANE RSEVLRKLSH LETISKPEPQ

                       2351
BEMITA_QJQ31013        S~
MYZUPE_XP_022181497    S~
TRIAVA_ACCase          NS
220O                   S~
319                    S~
419                    S~
520                    S~
619                    S~
```
